# Closely related *Campylobacter jejuni *strains from different sources reveal a generalist rather than a specialist lifestyle

**DOI:** 10.1186/1471-2164-12-584

**Published:** 2011-11-28

**Authors:** Eugenia Gripp, Daniela Hlahla, Xavier Didelot, Friederike Kops, Sven Maurischat, Karsten Tedin, Thomas Alter, Lüppo Ellerbroek, Kerstin Schreiber, Dietmar Schomburg, Traute Janssen, Patrick Bartholomäus, Dirk Hofreuter, Sabrina Woltemate, Markus Uhr, Birgit Brenneke, Petra Grüning, Gerald Gerlach, Lothar Wieler, Sebastian Suerbaum, Christine Josenhans

**Affiliations:** 1 Institute for Medical Microbiology, Hannover Medical School, Hannover, Germany; 2 Department of Statistics, University of Oxford, UK; 3 Institute for Microbiology and Epizootics, Freie Universität Berlin, Germany; 4 Federal Institute for Risk Assessment, Berlin, Germany; 5 Institute for Biochemistry and Biotechnology, Technische Universität Braunschweig, Germany; 6 Institute for Microbiology, University of Veterinary Medicine, Hannover, Germany; 7 Institute of Food Hygiene, Freie Universität Berlin, Germany

## Abstract

**Background:**

*Campylobacter jejuni *and *Campylobacter coli *are human intestinal pathogens of global importance. Zoonotic transmission from livestock animals or animal-derived food is the likely cause for most of these infections. However, little is known about their general and host-specific mechanisms of colonization, or virulence and pathogenicity factors. In certain hosts, *Campylobacter *species colonize persistently and do not cause disease, while they cause acute intestinal disease in humans.

**Results:**

Here, we investigate putative host-specificity using phenotypic characterization and genome-wide analysis of genetically closely related *C. jejuni *strains from different sources. A collection of 473 fresh *Campylobacter *isolates from Germany was assembled between 2006 and 2010 and characterized using MLST. A subset of closely related *C. jejuni *strains of the highly prevalent sequence type ST-21 was selected from different hosts and isolation sources. PCR typing of strain-variable genes provided evidence that some genes differed between these strains. Furthermore, phenotypic variation of these strains was tested using the following criteria: metabolic variation, protein expression patterns, and eukaryotic cell interaction. The results demonstrated remarkable phenotypic diversity within the ST-21 group, which however did not correlate with isolation source. Whole genome sequencing was performed for five ST-21 strains from chicken, human, bovine, and food sources, in order to gain insight into ST-21 genome diversity. The comparisons showed extensive genomic diversity, primarily due to recombination and gain of phage-related genes. By contrast, no genomic features associated with isolation source or host were identified.

**Conclusions:**

The genome information and phenotypic data obtained in vitro and in a chicken infection model provided little evidence of fixed adaptation to a specific host. Instead, the dominant *C. jejuni *ST-21 appeared to be characterized by phenotypic flexibility and high genetic microdiversity, revealing properties of a generalist. High genetic flexibility might allow generalist variants of *C. jejuni *to reversibly express diverse fitness factors in changing environments.

## Background

Intestinal infections by *Campylobacter jejuni *and *Campylobacter coli *are, jointly with *Salmonella enterica *infections, the most frequent bacterial causes of diarrheal diseases of humans worldwide [1]. *C. jejuni *causes the majority (> 83%) of human symptomatic *Campylobacter *infections [2]. *Campylobacter *ssp. are transmitted mainly by contaminated food of animal origin [3-8]. Animals are frequently infected persistently with Campylobacters and, in most cases, show no symptoms, contrary to the acute and self-limiting intestinal infection caused in humans. The cause of these discrepant outcomes of infection in different hosts is not clear, although previous work has suggested that host as well as bacterial factors may contribute [9-13]. Numerous *C. coli *and *C. jejuni *subtypes with similar morphology and biochemistry are found in different mammalian and bird hosts, with the possibility of zoonotic transmission to humans. In addition, *Campylobacter *species display extensive intraspecies variation. These properties require sophisticated molecular typing methods in order to differentiate species and subtypes, and to elucidate infection sources. Multi Locus Sequence Typing (MLST) analyses have proven to be a molecular method of choice and previously have provided clear evidence that genetically related strains of either *C. jejuni *or *C. coli *can colonize different hosts [13-19]. These strains frequently are part of large clonal complexes, whose members share most of the MLST alleles. However, MLST studies rely solely on the analysis of seven housekeeping genes, belonging to the core genome [16], and disregard the rest of the ~1.7 Mb genome [20], which includes virulence-associated genes as well as possible strain-specific genes or gene variants. Thus, the question remains open, whether different strain subtypes infecting humans differ from animal-colonizing and food-associated types within one closely related genetic grouping (such as an MLST sequence type).

Little is known about the mechanisms of host-specific colonization, virulence and pathogenicity factors of *Campylobacter *species. With few exceptions [21,22], genome sequences currently available in databases for *Campylobacter *species (see: NCBI „Microbial Genomes"; http://www.ncbi.nlm.nih.gov/genomes; [23,24]) have been derived from human isolates, therefore genome information about strains from other sources is still scarce. In order to complement these recent advances, it may be relevant to gather more information about host association and animal-associated strains and subtypes, some of which may be more prone to be transmitted or cause severe complications during human infections. Prophylactic measures are required to prevent transmission from food and animals to humans, which also require a better knowledge of strain-to-strain variability and adaptation potential to different hosts and environments such as food. This study was designed to characterize the genetic relationships and population structure of *C. jejuni *and *C. coli *currently prevalent in humans, domestic animals and food sources in Germany. Furthermore, a major aim was to establish the extent of diversity within dominant phylogenetic groups of *C. jejuni *from this collection, which are present in diverse hosts, such as ST-21, with regard to their different sources.

To that end, we collected 473 *C. jejuni *and *C. coli *isolates from humans, as well as animal (poultry, porcine, bovine), food, and environmental isolates between 2006 and 2010 in Germany. MLST analysis and extensive molecular typing of the isolates was performed, which confirmed *C. jejuni *ST-21 as one of the dominant "generalist" variants occurring in all sources. Further phenotypic characterization and whole genome analyses of closely related ST-21 *C. jejuni *strains from different sources provided little evidence for host or source-associated traits. Nevertheless, ST-21 isolates from different sources displayed a high genomic and phenotypic diversity and flexibility. Domestic circulating *C. jejuni *STs such as ST-21 appear to use mechanisms such as inter-strain recombination, metabolic and surface-associated genetic modules and contingency genes to permit a generalist lifestyle and the flexible expression of fitness factors in diverse environments.

## Results

### Multi Locus Sequence Typing of *C. coli *and *C. jejuni *isolates from diverse isolation sources in humans, animals and food sources

**Figure 1:**
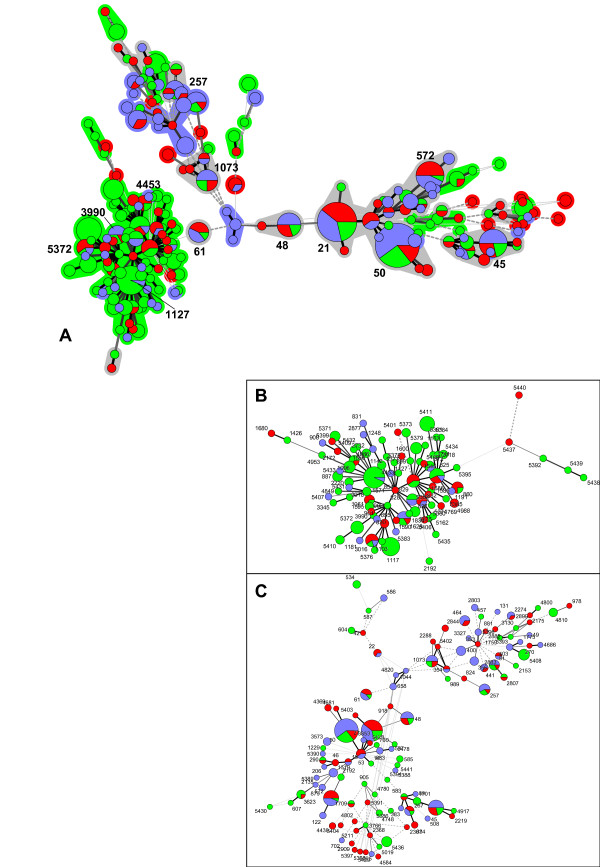
**Phylogenetic analysis of *Campylobacter *FBI-Zoo strain collection from humans, animals and food sources by MLST (Minimal Spanning Tree analysis-methods)**. A) Minimal spanning tree generated from MLST comparisons of all *Campylobacter *strains from the collection (Methods). B) Minimal spanning tree of *C. coli *strains. C) Minimal spanning tree of *C. jejuni *strains. Each circle represents one sequence type (all ST numbers indicated in B and C). In A), only dominant STs are indicated by numbers. Circles of increased diameter represent higher strain numbers within one ST. Different colours indicate strain source: blue-human; red-food; green-animal. The connecting lines between STs depict the number of allelic differences between them: one allele difference (black bold lines); two alleles difference (grey bold lines [A] or thin lines [B, C]); three alleles difference (grey hatched lines); more than three alleles difference (grey dotted lines). The phylogenetic group on the left of ST-61 represents all *C. coli *isolates, the phylogenetic tree expanding to the right of ST-61 comprises the *C. jejuni *strains. Clonal groupings (maximum two allele difference between neigbouring STs) in A) are indicated by colored shading around the circles and dominant STs within important groupings are designated with their respective ST numbers. The shading of clonal groupings is colored according to the majority isolation source. Grey shading indicates no predominance of source within the clonal groupings.

We collected 473 *Campylobacter *strains (173 *C. coli *and 300 *C. jejuni*) from animal hosts (chicken, pigs, cattle, wild birds, monkeys, pet animals), human patients, and food sources over a five-year period (2006 to 2010) in several geographically separate centers in Germany. Human patient samples were collected in three university hospitals (Hannover, Munich, Muenster); the majority of non-patient isolates were from domestic animals and food samples, while few strains originated from wild-living animals. Molecular typing of the whole strain collection was performed by MLST [15,25]. 197 different sequence types (STs; 112 for *C. jejuni *and 85 for *C. coli*) were identified, of which 57 were new STs not previously represented in the PubMLST database. These new STs include 37 that are novel combinations of previously known alleles, and 20 that included 19 novel alleles (further statistics, including separate analyses for *C. coli *and *C. jejuni*, in Table 1). The ST distribution was highly diverse, with 121 STs occurring only once, and the ten most common STs comprising only 43.7% and 48.0% of isolates for *C. jejuni *and *C. coli*, respectively. Of the 76 STs represented more than once, 22 were found in humans and, in addition, in either animals or food or in both of the latter, suggesting a host range consistent with zoonotic potential. All STs contained either only *C. jejuni *or *C. coli *strains. Only two alleles were found to occur in both species, *pgm*188, present in 4 *C. coli *and 2 *C. jejuni *strains, and *uncA*17, a very common allele present in 131 *C. coli *and 7 *C. jejuni *strains. Minimal spanning trees were generated from the MLST data illustrating the phylogenetic relationships of the strains (Figure 1). In confirmation and extension of previous results, MLST revealed dominant sequence types within the strains (Figure 1 and Additional File 1: Table S1). The dominant sequence types in the FBI-Zoo collection partially overlapped with dominant sequence types reported for *Campylobacter *samplings in the United Kingdom, the Netherlands, the U.S., Canada, Denmark and Finland (Additional File 2: Figure S2), which provide the majority of MLST types previously deposited in the public MLST database (Campylobacter PubMLST; http://PubMLST.org), and in some other countries. This result suggests that *Campylobacter *sequence types prevalent in many geographic areas of the world represent similar dominant strain types and clonal complexes. The five most dominant MLST sequence types for *C. jejuni *in the strain collection were ST-50, ST-21, ST-572, ST-45, St-48, and for *C. coli *ST-854, ST-1117, ST-5411, ST-825, and ST-1628. Many common MLST types, such as ST-21, ST-45, ST-48, were distributed rather evenly between strains isolated from domestic animal, human and food sources (Figure 1, Table 1). Some, mostly rarer, strain types were only found either in animals, food sources or in humans. *C. coli *strains were more closely related to each other than the *C. jejuni *strains (Figure 1), in concordance with previous studies [26,27].

**Table 1:** MLST analysis of FBI-Zoo *Campylobacter *strain collection

Number of strains	473
- *C. jejuni*	300

- *C. coli*	173

Source (*C. jejuni*/*C. coli*)	

human patients	134 (117/17)

Animals	210 (90/120)

Food	129 (93/36)

MLST	

Number of STs in dataset	197

- occurring in multiple strains	76

- singletons	121

novel STs	57

novel alleles	19

10 most frequent *C. jejuni *STs	no. of strains per ST (percent) strains from animals/food/human patients

ST-50	34 (11.3%) 9/5/20

ST-21	27 (9.0%) 6/11/10

ST-572	13 (4.3%) 1/7/5

ST-45	12 (4.0%) 3/4/5

ST-48	9 (3.0%) 2/3/4

ST-1073	8 (2.7%) 2/2/4

ST-61	7 (2.3%) 1/3/3

ST-257	7 (2.3%) 2/1/4

ST-5408	7 (2.3%) 7/0/0

ST-5411	7 (2.3%) 7/0/0

10 most frequent *C. coli *STs	no. of strains per ST (percent) strains from animals/food/human patients

ST-854	15 (8.7%) 14/0/1

ST-1117	11 (6.4%) 11/0/0

ST-5411	7 (4.0%) 7/0/0

ST-825	6 (3.5%) 4/2/0

ST-1628	6 (3.5%) 2/1/3

ST-829	5 (2.9%) 2/3/0

ST-3016	5 (2.9%) 1/3/1

ST-5372	5 (2.9%) 5/0/0

ST-5379	4 (2.3%) 4/0/0

ST-1556	4 (2.3%) 4/0/0

Standardized Index of Association (sIA)	

all strains	0.45

*C. coli *only	0.07

*C. jejuni *only	0.45

eBURST V3 analysis	

No. of groups (strains included)	19 (385)

singleton strains	88

The standardized Index of Association, (sI_A_) [28], was calculated for the complete set of allelic profiles and separately for *C. coli *and *C. jejuni *strains, respectively. sI_A _was 0.45 for the complete dataset and the *C. jejuni *dataset, indicating a limited amount of recombination not sufficient to completely destroy linkage between alleles. By contrast, the sI_A _for the *C. coli *dataset was 0.07, a value close to the expected value for linkage equilibrium (free recombination).

### Extended genetic pathotyping of *Campylobacter jejuni*

**Figure 2:**
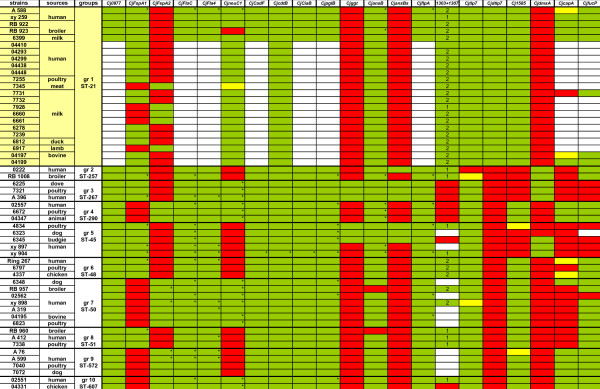
**Pathotyping scheme of 55 *C. jejuni *strains ordered according to STs**. Each of ten groups represents one ST and contains at least one strain from human, one strain from animal and one strain from food sources. The distribution of putative virulence- and host-associated genes was tested by PCR (primer combinations in Table 2). The presence of genes Cj1305 and Cj1306 was detected using a combination of primers in Cj1303 and Cj1307, by PCR product length: 1 indicates the presence of just one gene, either Cj1305 or Cj1306, and 2 indicates the presence of both genes. Color-coding: green: gene present; red: gene not present; yellow: weak PCR product; white: not tested. Gene copies marked by asterisks were additionally sequenced to identify interstrain polymorphisms using Sanger sequencing.

Due to the restriction of the Campylobacter MLST system to seven housekeeping genes, potential host-specific genes, genes providing strain-specific means of enhanced survival and transmission, or predictive markers for infection-associated severe disease may only be detected by looking at an enhanced set of proposed virulence genes, or at the complete genomes of strains. We therefore selected a subgroup of *C. jejuni *strains (91 strains; Additional File 2: Table S2) from the FBI-Zoo collection that contained representative isolates of all dominant MLST sequence types, and included strains from all major available isolation sources (human, diverse animals, food materials) for all dominant STs. Each strain in this subset was subjected to PCR-based typing to detect the presence or absence of a panel of 18 genes previously proposed to be involved in pathogenesis, metabolism or host specificity (Additional File 2: Table S2). Three additional genes were included for the typing of 55 *C. jejuni *strains belonging to ten different STs (Figure 2). A few tested genes were present in all strains, such as *Cj0977*, *flaC*, *flaA*, *cadF*, *pglB*, and *flpA *(proposed adhesin [29]). Most of the tested genes were only detectable in a subset of the strains, including *ggt *(gamma-glytamyl-transpeptidase), secreted *ansB *(asparaginase), *dmsA*, *cdtB*, *fucP*, *ciaB*, *capA *(presumed adhesin [29]) and *neuC1 *(sialyltransferase) genes [12,30-32]. Both the *ggt *gene and the secreted *ansB *(*ansB_s_*) allelic variant were previously proposed to provide niche or host specificity [12,31]. Interestingly, the proportion of strains possessing *ggt *or *ansB_s _*genes was overall very low in our strain collection (< 8%). In addition, in isogenic MLST groups containing strains from all three major isolation sources (human, food, animal), the presence of the *neuC1*, *ggt *or *ansB_s _*genes did not correlate with isolation source. Nor did the presence of any other gene in the pathotyping matrix (Figure 2; Additional File 2: Table S2) correlate with isolation source. However, close correlation with MLST type was detected for the proposed virulence genes *fspA1 *and *fspA2 *[33], except for ST-21, suggesting that these gene types are usually coinherited in phylogenetically closely related strain subtypes. This result corroborates recent data for *fspA1 *obtained from Finnish strains [34]. Overall, taking into account phylogenetic relatedness of strains within ST groups, no correlation of presence of individual genes with the isolation source was observed. Additional genes involved in metabolic functions and iron uptake (*exbB1*, *ceuB-E*, *crfA*) were tested for presence or absence in a subset of the 91 strains from different STs and isolation sources and did not show a host-specific distribution (not depicted). For some of the genes which were present in all or most strains (*cdtB*, *flaC*, *flaA*, *ciaB*) and some non-ubiquitous genes (*neuC1*, *dmsA*, *tlp7, ggt, fspA1*, *fspA2*), we performed sequencing of the genes on a subset of PCR-typed strains (60 strains from 39 MLST sequence types; asterisks in Figure 2). Sequence diversity between the strains was found in these genes to a different extent (data not shown). In summary, none of the tested genes or allelic variants taken singly or in combination were correlated with isolation source.

### Evidence of phenotypic differences between *C. jejuni *ST-21 strains from different sources

**Figure 3:**
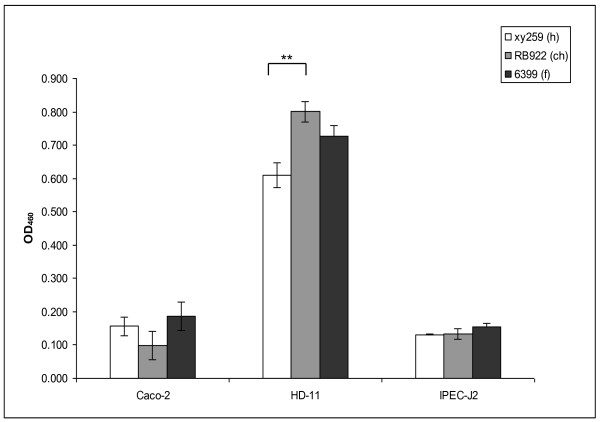
**Phenotypic difference in adherence of three ST-21 *C. jejuni *strains from human, animal and food source to mammalian cells**. Human colonic epithelial cells (CaCo-2), chicken macrophage-like cells (HD-11) and porcine intestinal epithelial cells (IPEC-J2) were infected in triplicates with biotin-labelled bacteria at an MOI of 200 and coincubated for 1 h (Methods). Each bar represents the mean ± standard deviation number of adherent bacteria of one *C. jejuni *strain from triplicate measurement in one representative experiment. Statistical differences in adherence were evaluated using a Student's *t *test. A p value of < 0.05 was considered statistically significant (* p ≤ 0.05, ** p ≤ 0.01, *** p ≤ 0.001). h = human, ch = chicken, f = food.

**Figure 4:**
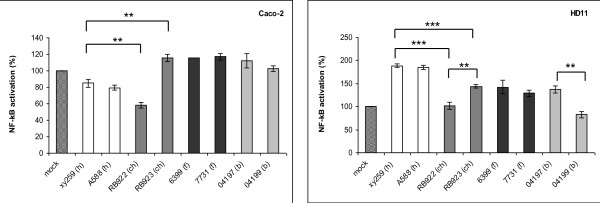
**Differential NF-κB activation or inhibition by various ST-21 *C. jejuni *strains in mammalian cells**. Stably transfected Caco-2 cells (A) and HD-11 cells (B) containing a NF-κB-luciferase gene fusion were coincubated with 8 different *C. jejuni *strains (from human (h), chicken (ch), bovine (b) and food (f) source) at an MOI of 50 in triplicates and coincubated for 1 h. The values are depicted in percent relative to the mock-infected sample, which was set to 100%. One representative experiment of four is displayed. Statistics was performed using a Student's *t *test. A p-value of < 0.05 was considered statistically significant (* p ≤ 0.05, ** p ≤ 0.01, *** p ≤ 0.001). Selected statistical evaluation of differences between strains from one source or between two different sources is shown.

**Figure 5:**
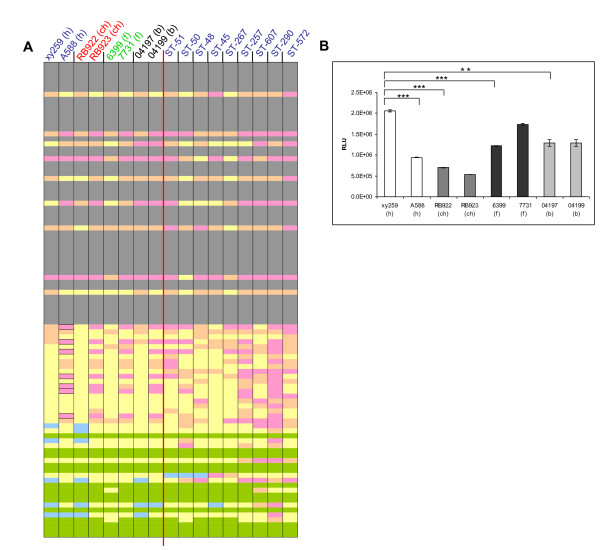
**Differences between ST-21 *C. jejuni *strains in metabolic activity and energy levels**. (A) Carbon source utilization by eight ST-21 strains (left panel) from different sources (h = human; ch = chicken; b = bovine; f = food) and nine human-derived strains (from left to right: xy898, R267, xy904, A396, 222, 02551, 02557, A412 and A599) of different STs (right panel) were tested at 42°C using BioLog Phenotype Microarray PM1 plates (area values). Grey color indicates no or only weak substrate utilization for all strains (all area values below 800) and green indicates high substrate utilization for all strains (all area values above 800). The colors pink, yellow, beige and blue indicate strain-specific utilization: pink-zero utilization, beige-< 1,000, yellow-< 9,000, blue-> 9,000. The six bordered rectangles on the left panel (from top to bottom, second from left column) indicate the substrates L-galactonic acid-g-lactone, tyramine, m-hydroxy-phenylacetic acid, p-hydroxy-phenylacetic acid, glycyl-L-aspartic acid, glycyl-L-glutamic acid and glycyl-L-proline, which are not used by some strains (phenotypic variant 1). PM1 experiments with ST-21 strains were performed at least three times independently on different days. Shown for each substrate are the mean area values of three independent experiments performed at 42°C for 30 h incubation time. PM1 experiments for other STs were performed either once or twice (mean values shown for ST-45 and ST-267 strains) at 42°C. (B) Differences in intracellular ATP levels (energy harvest) of eight ST-21 strains from different sources. Statistics was performed using a Student's *t *test. A p-value of < 0.05 was considered statistically significant (* p ≤ 0.05, ** p ≤ 0.01, *** p ≤ 0.001).

The data described above suggested that the identification of any genes, gene variants or traits potentially providing host specificity to *C. jejuni *might require more sophisticated methodological approaches. We therefore decided to perform in-depth investigations of the phenotypic and genetic traits of eight selected *C. jejuni *strains from one single exemplary MLST sequence type (ST-21), a common and dominant ST worldwide. The selected ST-21 strains (xy259, A588, RB922, RB923, 6399, 7731, 04197, 04199; Figure 2) were isolated from human patients, domestic animals (cattle, chicken) and food sources; PCR typing of strain-variable genes (Figure 2) had provided evidence that some genes differed between these strains. This prompted us to first address the question whether these strains show any other phenotypic variation which would warrant further characterization with regard to the diversification of closely related strains. We tested the phenotypic variation of these strains using the following criteria: metabolic variation (Biolog PM1 plate, testing a panel of carbon-providing substrates including sugars and some amino acids; Additional File 2: Table S3), energy harvest, motility, morphology, protein expression patterns and cell interaction. Some of the experiments were performed at 42°C and at 37°C. All of these freshly isolated strains possessed very high motility except for the milk-derived strain 6399. The bacterial morphology in transmission electron microscopy was very similar. Cell interaction of the strains was comparatively tested in adherence assays with the human intestinal epithelial cell line CaCo2, the chicken macrophage-like cell line HD-11, the IPEC-J2 swine intestinal epithelial cell line (Figure 3) and human epithelial HeLa cells (not shown). In addition, proinflammatory cytokine production (data not shown) and cell activation were tested for eight ST-21 strains on the same cell lines containing an NF-kB-driven luciferase reporter (Figure 4). The tested ST-21 strains showed differences in cell adherence, cytokine production and NF-kB activation (Figure 3; Figure 4), indicating bacterial phenotypic differences. Next, we used rabbit-derived and chicken sera reactive against *C. jejuni *in order to compare the patterns of immunogenic proteins between different strains of the same sequence type in Western blots. Differential protein patterns were detected in closely related strains when cultivated at both 37°C and 42°C (data not shown). Likewise, MLST-isogenic strains differed in their metabolic properties and energy harvest as determined by Biolog analysis and intrabacterial ATP levels of eight ST-21 strains (Figure 5A, C, Additional File 2: Table S3). Interestingly, using the Biolog characteristics, the group ST-21 strains could be largely subdivided into two phenotypic variants with characteristic patterns of properties. These two variants (biotype 1 and biotype 2) were represented with equal frequency in the eight strains (two strains each from human, chicken, bovine and milk sources) tested by Biolog, and did not show any correlation to a specific host or source. The common characteristics of half of these strains (biotype 2) were the metabolic use of three specific glycyl-L-dipeptides (glycyl-L-glutamic acid, glycyl-L-aspartic acid, glycyl-L-proline), L-galactonic acid g-lactone, tyramine, m/p-hydroxy-phenylacetic acid (Figure 5A, Additional File 2: Table S3), while the biotype 1 strains did not use those. These metabolic trait differences had not been described before for closely related *C. jejuni *strains. These results were very reproducible and not temperature-dependent (expressed by the respective strains at both 37°C and 42°C). When nine additional strains from other STs (human sources only, Figure 5A; Additional File 2: Table S3) were tested in the same Biolog system, distinct metabolic fingerprints were observed for each ST. Surprisingly, some of the tested strains also displayed increased electron chain activity with one hexose sugar (D-mannose) and one phosphate sugar (D-fructose-6-phosphate). In summary, these results provided clear evidence for phenotypic differences between ST-21 strains, with no indication of host specificity.

### Complete genome analysis of genetically closely related *C. jejuni *ST-21 strains reveals interstrain variation

**Figure 6:**
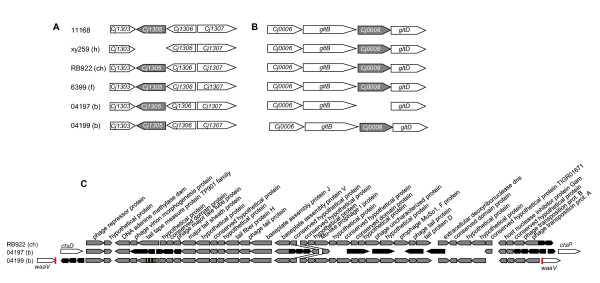
**Examples for gene loss or gain in complete genome sequences of five ST-21 *C. jejuni *strains**. (A) Loss of ortholog to gene *Cj1305*, which belongs to the 617 family located in the flagellin glycosylation gene cluster, in the human strain xy259. (B) Loss of ortholog to gene *Cj0008 *in 04197 nestled in between putative pyridine nucleotide-disulphide oxidoreductase large subunit gene *gltB *and small subunit gene *gltD*. (C) Common phage insertion in three ST-21 strains. The flanking genes of the phage insertion site are indicated by white arrows in the strains 04197 and 04199. Grey arrows show phage genes similar in all strains, black arrows indicate unique phage genes. The phage is related to CJIE1 in the whole genome sequence of *C. jejuni *RM1221 [20].

**Figure 7:**
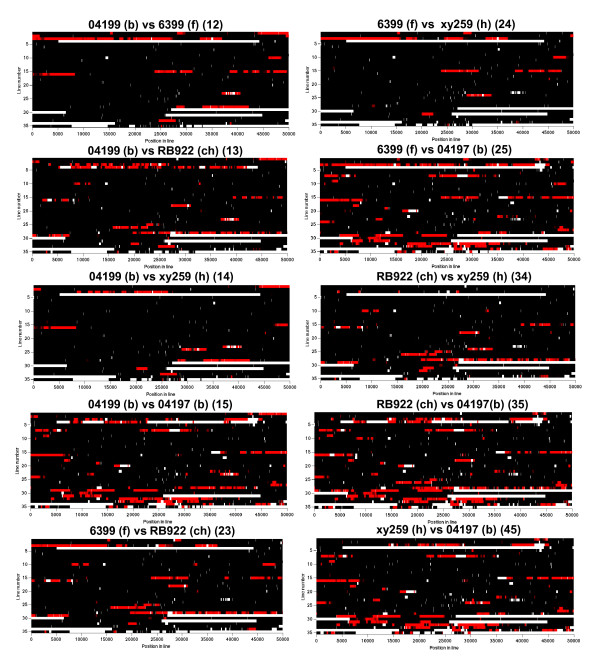
**Pairwise nucleotide comparisons of five complete ST-21 *C. jejuni *genome sequences**. The comparisons were performed using a Bayesian statistics model [25], with black representing identical sequence stretches, white representing non-aligned (unrelated or missing) sequences, and red areas indicating imported/recombined stretches of sequence (h = human; ch = chicken; f = food; b = bovine). The two-digit number behind each header indicates the strain comparison (each digit corresponds to one strain), equivalent to Additional File 3: Table S10. The intensity of each color represents statistical uncertainty. One horizontal line (line number of y-axes) is equivalent to 50,000 kilobases (position in line of the x-axes corresponds to the basepair count). For all alignments, strain 04199 (largest genome, strain number 1 in genome alignments) was used as a reference genome.

In order to test the hypothesis of a genetic basis for the observed phenotypic diversity within the ST-21 group, whole genome sequencing was performed for five ST-21 strains from bovine, chicken, human and food sources (strains: xy259, RB922, 6399, 04197, 04199; see Additional File 2: Table S4 for genome statistics). This approach was also aimed at answering the question, whether the observed phenotypic diversity was due to unique genomic events which could not be identified with pathotyping of known genes, and whether it was somehow correlated with association to a specific host. The genome sequences revealed relatively little variation in gene content (macrodiversity; Figure 6; Additional File 2: Table S5) but a high level of polymorphism within shared genes, contingency genes and intergenic regions (Figure 7). Those included single nucleotide (SNP) and clustered nucleotide polymorphisms (CNP) (Additional File 2: Figure S4 and Table S6). Sequence length differences were mostly found in intergenic regions (Additional File 2: Table S8). Four of the five sequenced strains had acquired phage gene clusters (Figure 6; Additional File 2: Tables S5, S9). Four selected phage genes were tested by specific PCR for their presence in a wider array of ST-21 strains and found to represent single events (Additional File 2: Table S9), uncorrelated with any host or source. The human isolate xy259 possessed a deletion in a gene responsible for surface or flagellar glycosylation (gene 617 family, gene *Cj1305*-like; Figure 6), which was not found in other human strains. The bovine strain 04197 lacked the gene equivalent to *Cj0008 *(gene designation of strain 11168), which is a gene of unknown function in a metabolic gene cluster relating to a pyridine nucleotide-disulphide oxidoreductase. The allelic diversity between the strains comprised a large number of synonymous and non-synonymous nucleotide substitutions in coding regions (Additional File 2: Table S6), many of which originated from homologous recombination, and some deletions and insertions in both coding and intergenic regions of all strains (Additional File 2: Table S8). Repeat length differences probably originating from slipped strand mispairing mutagenesis were evident in all strains. This concerned eight genes of the glycosylation gene clusters leading to frameshifts in coding regions. We confirmed these repeat length differences in a larger number of ST-21 strains using Sanger sequencing (Additional File 2: Table S7). By this extended sequence determination, the repeat length differences obtained by 454 sequencing were confirmed, but none of the tested nucleotide polymorphisms was stably associated with the source of the strain. Only a limited number of single nucleotide polymorphisms and repeat differences could be investigated by this labour-intensive approach. Aligning the five genome sequences (Methods; Figure 7, Additional File 3: Table S10) revealed features of genome plasticity in *C. jejuni*, with a highly conserved overall synteny of the genomes. We detected that frequent homologous recombination events had happened since the divergence of the genomes from a common ancestor (Figure 7, Additional File 2: Table S6, Additional File 3: Table S10), with an average imported length of 1437 bp (maximum length of CNP up to ~ 15 kbp). The genome regions containing clustered polymorphisms caused by recombination are summarized for all five ST-21 genomes in Additional File 2: Table S6. They contain genes of various functional categories (according to the COG classification). None of the functional categories showed significantly higher percentages for having undergone recombination than the average of all functional categories (Additional File 2: Figure S3). When the ST-21 genomes were aligned to previously sequenced *C. jejuni *genomes from unrelated phylogenetic groups (e.g. of strains RM1221 [22] and 81 176 [30]) in addition to nucleotide polymorphisms, notable differences in gene content were observed (mainly genes involved in metabolism, glycosylation, and restriction-modification genes; not shown).

### Correlating phenotypic variation with genetic differences in ST-21 strains

Both genotypic and phenotypic differences were observed for ST-21 and other *C. jejuni *strains, and the genotypic differences might be a basis for a potential adaptive phenotype. From the Biolog assays, there was no evidence for a correlation of the two different metabolic biotypes within ST-21 with any specific source. Nevertheless, it is worthwhile investigating the gene variants potentially providing phenotypic differences, including those of metabolic capacities detected in the Biolog assay. We next asked if genetic polymorphisms within ST-21 that had been identified by the genome sequence analysis could be correlated with the metabolic biotypes. As already described previously, strains of the ST-21 lineage are able to use fucose, due to the presence of a genomic island [35,36]. As a proof of concept, use of fucose was confirmed for all tested ST-21 strains (which possess the fucose island in their complete genome sequences) using the Biolog system (Figure 5A). Within the other tested STs, ST-45 and ST-267 did not display fucose utilization, as expected from previous studies [35]. One of the ST-21 strains selected for whole genome sequencing, the bovine isolate 04199, belonged to metabolic biotype 1 and did not metabolize the three dipeptides, tyramine, and m/p-hydroxy-phenylacetic acid. Although several genetic polymorphisms found in the 04199 genome were investigated in all ST-21 strains belonging to biotype 1, and compared with the corresponding loci of biotype 2 strains, a specific genetic variant which could be responsible for the metabolic phenotype was not identified. Several genes and intergenic regions of potential relevance for metabolic activities varied between ST-21 strains (mostly sequence polymorphisms or small insertions/deletions; see also Additional File 2: Tables S6, S8). These included two peptidase genes [*Cj0805*-like, *Cj0703*-like], *msrA *[methionine sulfoxide reductase subunit], *cmeB *[efflux pump subunit] (both genes contain polymorphisms in strain 04199), *sdhA *[novel type fumarate dehydrogenase subunit; [37]]*, frdA *[fumarate dehydrogenase subunit]*, dcuB *[C4-dicarboxylate transporter] (three genes contain polymorphisms in strain RB922)*, ansB *[asparaginase], and *argS *[arginyl-tRNA synthetase] (the latter gene distinct in strain 6399). These allelic or intergenic differences were resequenced or tested with allele-specific primers for further isolates of ST-21 and other STs from various sources. None of them could be affirmed to be source-associated or related to metabolic biotype for carbon sources. In addition, RNA-based real time PCR was performed for six ST-21 strains (five of which were the genome-sequenced isolates), two of biotype 1 and three of biotype 2 (04199 [1], A588 [1], xy259, 6399, RB922, 04197), to elucidate whether genes were differentially transcribed, in particular genes of global regulators. These analyses yielded clear differences in transcript abundance between the strains (for *rpoN*, *flgR *and *luxS *regulators) which were uncorrelated with metabolic biotype or source (not shown). Most conspicuously, strain 6399 (milk isolate) appeared to have a largely reduced *rpoN *transcript amount, which also seemed to negatively affect *luxS *transcript, and matched the low observed *in vitro *motility of this strain.

### Chicken colonization by two genetically closely related ST-21 *C. jejuni *strains from human and chicken

**Figure 8:**
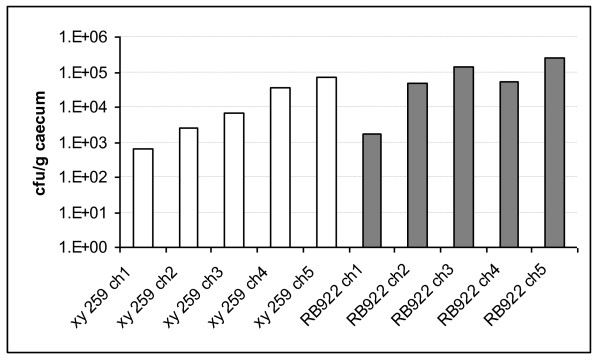
**Chicken colonization of human-derived and chicken-derived *C. jejuni *ST-21 isolates**. Real time PCR was performed on DNA isolated from caecal tissue of chickens after 14 d of infection (methods). Colony-forming units per g caecum were calculated from standardized bacterial DNA samples equivalent to a specific number of bacteria. White bars indicate chicks infected with human-derived strain xy259 (animals designated xy259 ch1 to ch5); grey bars indicate chicks inoculated with chicken-derived strain RB922 (animals RB922 ch1 to ch5).

Chicken colonization studies have helped previously to determine whether certain *C. jejuni *variants were infection-competent, including studies that tested strains with presumed adaptation to other hosts that were no longer able to infect chickens [21]. We infected three weeks-old white leghorn chicks with two of the genome-sequenced closely related ST-21 strains, xy259 (human) and RB922 (chicken). All inoculated animals were colonized by both test strains, which had been verified in advance to possess full motility, since motility is a major prerequisite for successful colonization of campylobacters in any host and subject to strong phase variation mechanisms [38]. During the two-weeks' infection period, the chicken-derived strain colonized chickens slightly better (up to one log higher bacterial loads per animal) than the human-derived strain (Figure 8). However, the human strain was fully competent to colonize all animals persistently.

## Discussion

In order to curb the high numbers of human *Campylobacter *infections, zoonotic transmission to humans needs to be reduced. Hence, the identification of specific groups of strains or strain properties which are associated with the colonization of humans or specific animals may help to provide targets for such measures. In the present study, an in-depth genetic and phenotypic characterization of a diverse array of *Campylobacter *strains was initiated, including in particular closely genetically related strains of ST-21 isolated from diverse habitats and isolation sources.

From 2006 to 2010, *Campylobacter *isolates were collected in the framework of the FBI-Zoo network from humans, diverse animals and food sources in Germany. The complete collection of 473 strains was subjected to MLST analysis, making this one of the most comprehensive MLST analyses of *Campylobacter *from one country outside of the UK. Approximately one third of *C. jejuni *and one fourth of *C. coli *isolates belonged to five dominant STs, each representing at least 3% of the isolates. Many dominant STs are also very prevalent in other geographical regions (e.g. *C. jejuni *STs 45, 48, 50, 21, 267; see also Additional File 2: Figure S2); as in other European regions, ST-574, which is prevalent in New Zealand (http://PubMLST.org), was not identified in Germany. For *C. jejuni*, strains of all dominant STs were isolated from both humans and animals/food samples, indicating a broad host range and zoonotic potential. In contrast, approximately two thirds of *C. jejuni *strains were very diverse and represent rare STs, so that the low number of isolates per ST made it impossible to draw valid conclusions about their host association. These and previous data suggest that all dominant STs found in a variety of sources have a "generalist" phenotype, enabling them to colonize both humans and domestic animals. From these hosts, they can then be transmitted to humans either directly or via food. A previous whole genome-based study including 95 strains [16] suggested, that a host-specific *Campylobacter *subtype, or host-specific *Campylobacter *genes, may not exist, particularly in the dominant MLST sequence types, which are mainly derived from domestic sources that may have evolved under conditions of ample recent admixture [26]. This does not rule out that some of the rarer sequence types, such as isolates from wild animals which rarely mix with other niches, have irreversibly adapted to a single host species. Evidence for fixed host adaptation in a rare ST has been provided recently for sequence type ST-3704 [21,39], a *C. jejuni *variant from bank voles, which failed to colonize chickens. While strong evidence of admixture between *C. coli *and *C. jejuni *has been presented before [26], and is supported by the existence of alleles occurring in both *C. jejuni *and *C. coli*, the overall frequency of such shared alleles was very low in our collection (see also Additional File 1: Figure S1). Calculations of the standardized index of association and other tests for recombination were in agreement with previous studies demonstrating an important role of recombination in shaping the population structure of *Campylobacter *species. Notably, the sI_A _of *C. coli *was much lower than that of *C. jejuni*, suggesting more efficient exchange of alleles between *C. coli *strains than *C. jejuni *strains.

*C. jejuni *and *C. coli *have been shown to be versatile and diverse species, harbouring numerous genes related to host interactions or metabolism that are not present in all strains, or are subject to phase variation [20,30,40-42]. We have performed an extensive analysis of such genes in a large subset of our collection, and additional reference isolates. 91 *C. jejuni *strains were studied for the absence/presence and sequence variability of 18 genes with putative roles in phenotypes relevant to host interaction. These included genes coding for surface proteins, LOS, protein glycosylation and metabolic functions which have been suggested to play an important part in niche- or host-associated colonization abilities [43,31,45].

The present data have not provided evidence that any of the genes tested was robustly associated with isolation source. By contrast, pathotyping profiles were in many cases closely correlated with phylogenetic grouping by sequence types, suggesting that *Campylobacter *have diversified their complements of in vivo essential genes. Apparently, the host interaction gene complement of dominant STs was selected to provide equal fitness advantages in both domestic ("food") animals and humans, permitting strain circulation between humans and domestic animals, making them "generalists". These generalist strains could then make use of different genetic features including reversible frame-shifting of contingency genes, intra-genomic and inter-genomic recombination to fine-tune their genetic and phenotypic capacities, which may benefit their survival in individual hosts or changing environments without preventing their generalist adaptation potential.

ST-21 is one of the most prevalent sequence types of *C. jejuni *present in a broad range of hosts [14,15,17-19,46]. We have selected ST-21 to analyze the genetic and phenotypic diversity of *C. jejuni *within a group of closely related isolates, which appear to have generalist properties with respect to domestic hosts. Phenotypic characterization and metabolic profiling was performed for eight ST-21 isolates from different sources (two strains each from human, chicken, bovine and milk sources), and the genomes of five of these strains were sequenced. Metabolic profiling identified two characteristic biotypes within ST-21, with distinctive differences in their ability to metabolize certain carbon sources, including some dipeptides. Similar to the pathotyping results, none of these biotypes was associated with a specific isolation source, suggesting that both biotypes are compatible with a generalist lifestyle. In addition to differences in their metabolic capacities, the same strains also showed phenotypic diversity in their interaction with eukaryotic cells, independently of their isolation source. A further nine human *C. jejuni *strains from other rare and frequent STs (all isolated from humans) that were compared using the Biolog system showed metabolic fingerprints distinct from each other and also different from all ST-21 strains, revealing the metabolic fingerprint as a distinctive feature between and even within phylogenetic subtypes of the species. However, our study did not provide any evidence for a common metabolic fingerprint of human-derived strains.

The genome analysis of five ST-21 strains confirmed their high genetic relatedness, with long stretches of complete sequence identity and generally conserved synteny. However, pairwise and multiple comparisons of all sequences provided evidence of recombination that has caused the exchange of chromosome fragments of varying length, up to ~15 kbp. These genome mosaicisms are reminiscent of the situation in the human gastric pathogen *Helicobacter pylori*, where genome comparisons of sequentially isolated strains from one single individual have yielded very similar mosaic patterns [47] to the ones observed here. In particular, the average length of imported fragments in the ST-21 genomes was 1,437 bp, between two and six times more than a previous estimate based on MLST data only [48], which could reflect the fact that MLST genes are under strong purifying selection [49]. The import length is about 3-4 times longer than the average length of imports calculated from *H. pylori *genome comparisons (394 bp, [47]) with the same analytical model. Mutation and recombination were estimated to have occurred at similar rates, but recombination introduced 21.5 times more polymorphisms than mutation as it often substitutes several sites simultaneously [50]. Despite the strong evidence for recombination, the gene content in the five ST-21 genomes was very highly conserved. Only few strain-specific or unique genes were identified, most of which were part of phage gene clusters. These phage acquisitions represented almost the entire set of gene content differences between the sequenced ST-21 strains. Some phage genes were so far unknown; three of the phages (phage clusters 1, 2 and 7) were similar to previously described *Campylobacter *phages (CJIE1, CJIE3, CJIE4 families; [23,22,51,52]. The acquisition of some strain-specific genes appeared to be unique events and not related to the strain source or host. Yet, these closely related strains with identical MLST sequence types displayed substantial polymorphism ([20]; and Additional File 2: Table S7), largely caused by extensive inter-strain recombination and contingency genes. This corroborated earlier results from microarray comparisons which claimed that *C. jejuni *strains possess a high level of genome diversity, even within a single sequence type [53,54]. Extended testing for variant alleles of certain candidate genes in other dominant or rare STs or in specific groups of strains from specific host or source did not reveal any fixed host relation. Some of these variants may indicate a pressure for phenotypic diversification, for example due to bacteriophage resistance, or might still affect fitness in specific hosts or under certain colonization conditions. We propose that the observed microdiversity may allow these bacteria to flexibly and reversibly adapt to diverse habitats and to propagate their generalist phenotypes. Experimental infection of two genome-sequenced ST-21 strains in chickens revealed that a ST-21 strain isolated from a human was fully competent to colonize chickens, albeit to a slightly lower density than a chicken isolate inoculated in a parallel experiment. This suggests that no general colonization barrier for human-derived ST-21 strains exists in chicken. Extensive genetic analyses of 95 whole genomes of *C. coli *and *C. jejuni *(43 *C. jejuni *genomes) have recently revealed that genetically distantly related isolates show considerable variability in gene content [16]. The authors of this study, however, did not find evidence of statistical association between gene content and type of host. Additional work suggested that the observed genetic diversity between *C. jejuni *isolates were instead closely associated with phylogenetic origin of the strains [34]. In a recent study, a human *C. jejuni *strain was inoculated in parallel into outbred chickens and inbred genetically modified mice [55], resulting in a much lower variation in the output pool from the mice compared to that of chickens. In a second experimental system of strain diversification, one specific *C. jejuni *variant in a contingency gene was strongly enriched by serial mouse passage [56]. This may suggest that, in contrast to a host population of low genetic variability such as inbred mice, *Campylobacter *requires genetic flexibility in a host population consisting of phenotypically and immunologically diverse individuals, including factors such as resident microbiota and bacteriophage abundance. Given these findings, it seems plausible that abundant *Campylobacter *types profit in many different ways from their established genome flexibility, even within a single host species.

## Conclusions

While our findings do not rule out the existence of phenotypic and genotypic determinants of *Campylobacter *tissue tropism and host specificity, our results from ST-21 genomic and phenotypic comparisons rather support a view where all ST-21 strains are characterized by a generalist genome, phenotype and lifestyle, which support their ability to colonize both humans and food animals. The same might be true for other dominant *C. jejuni *STs and should be investigated further. This view is also supported by an extended MLST study of *Campylobacter *isolates in the UK [57]. These frequent types may possess a clear fitness advantage under diverse conditions. We propose that future efforts should be directed towards defining the molecular determinants of this phenotypic flexibility and generalist capacity, which might be useful targets for interventions into the transmission cycle, and hence ultimately reduce *Campylobacter*-associated diseases. For their high numbers of human infections caused world wide, and their very broad transmission capacities, the dominant *Campylobacter *STs deserve intensive attention. If the hypothesis of generalist and specialist strain types can be confirmed in larger *Campylobacter *strain collections, it would suggest to study host-specificity or the predictive value of molecular subtyping methods separately for the dominant and the rare ST groups.

## Methods

### Bacterial strains and culture conditions

The 473 recent strains (2006 to 2010) from the FBI-Zoo collection were collected in different regions in Germany (Additional File 1: Table S1) and from different hosts and food sources by members of the FBI-Zoo research network http://www.fbi-zoo.net. Additional reference strains of different sequence types were included in some of the analyses, which increased the number of analyzed strains to 492 (Additional File 1: Table S1). In some instances, Preston broth (Oxoid, Wesel, Germany) was used for bacterial enrichment, and Karmali or Skirrow Agar (Oxoid) for primary cultivation of *Campylobacter *spp.. All *Campylobacter *strains were routinely grown at 37°C or 42°C under microaerobic conditions (10% CO_2_, 5% O_2_, 85% N_2_) in vented jars on blood agar plates (Blood Agar Base II; Oxoid), supplemented with 10% defibrinated horse blood (Oxoid) and antibiotics (10 mg/liter vancomycin, 2,500 U/liter polymyxin B, 5 mg/liter trimethoprim, 4 mg/liter amphotericin B), or on thioglycollate agar (Oxoid). For DNA preparations, coincubation assays, lysates preparations and phenotypic arrays bacteria were grown on plates for approximately 24 h before harvest. For some applications, bacteria were incubated in air-tight jars using an atmosphere generated by Oxoid CampyGen or Merck Anaerocult C sachet (Merck, Darmstadt, Germany) catalysts.

### Cell types, cell culture conditions and cell coincubation with *Campylobacter *strains

Different cell lines were used for cell coincubation and adherence assays. The human colon adenocarcinoma cell line Caco-2 (ACC 57 and 169, German Collection of Microorganisms and Cell Cultures Braunschweig) was grown in Dulbecco's MEM supplemented with 10% [v/v] fetal bovine serum (FBS). The porcine intestinal epithelial cell line IPEC-J2 [58] was grown in Dulbecco's MEM and Ham's F-12 (1:1 mixture) supplemented with 5% [v/v] FBS, and the HD-11 chicken macrophage-like cell line was maintained in IMDM + GlutaMAX-I (Gibco, Darmstadt, Germany) supplemented with 10% [v/v] FBS. Caco2, HD-11 and IPEC-J2 cell lines stably transfected with the firefly luciferase gene were generated by K. Tedin and continuously cultured in the presence of puromycin (5 g/L). All cell lines were routinely kept at 37°C in a 5% CO_2 _humidified atmosphere.

For the cell adherence assays, Caco-2, IPEC-J2 and HD-11 cells were seeded at 2 × 10^4 ^cells per well in a 96 well plate and grown to near confluency. The cell medium was replaced prior to the coincubation, 1 h before the addition of bacterial cells. The bacterial strains were prepared as follows: bacteria were harvested from plates with a sterile cotton swab and resuspended in 2 ml sterile cold PBS, after that all steps were performed on ice. 2 × 10^8 ^bacteria were washed 3 times (5 min, 5,000 × g, 4°C) in PBS, gently resuspended in 450 μl PBS and surface-biotinylated by adding 125 μg EZ-link-TM-Sulfo-NHS-LC-Biotin (Thermo Scientific, Rockford, IL, USA). The subsequent incubation took place at RT in the dark for 50 min. Afterwards the bacteria were washed 3 times and resuspended in 200 μl cell culture medium. Before adding the bacteria to the cells, the bacteria were warmed to 37°C, added to the cells to an MOI of 50 and 200 bacteria per cell, and centrifuged for 5 min at 200 × g to ensure bacteria-host cell contact. The subsequent coincubation lasted 1 h, before 2 × washes with PBS were performed to remove the non-adherent bacteria. The following fixation step of the cells was initiated by removing the medium and by adding 100 μl of 2% paraformaldehyde (in 100 mM potassium phosphate buffer, pH 7.0) for 2 h at RT. After fixation, the wells were washed 3 times with PBS + 0.1% glycine and incubated in a blocking solution (PBS, pH 7.0, 10% FBS) for 1 h, followed by 5 times washing (PBS, pH 7.0, 0.05% [v/v] Tween 20). The cell layers together with adherent bacteria were incubated for 1 h with neutravidin-HRP-conjugate (in PBS pH = 7.0 with 10% FBS [v/v], (1:5000); Perbio Science, Germany). After 7 times washing (PBS, pH 7.0, 0.05% [v/v] Tween 20), the wells were incubated for 30 min with 100 μl TMB substrate (Perbio Science, Germany). The resulting colour reaction was stopped by adding 50 μl 1 M phosphoric acid and measured at 450 nm (reference filter 540 nm) in a microplate reader.

For the cytokine release assay (human IL-8), Caco-2 and Hela cells were seeded at 1.5 × 10^5 ^cells in 1 ml of medium per well in a 24 well plate and incubated, until a confluency of 80% was reached (app. 24 h). The medium was replaced before the addition of bacteria. The cell monolayers were infected with *C. jejuni *strains at an MOI of 10, centrifuged 10 min at 300 × g and coincubated for 4 h, before the supernatants were harvested. After a centrifugation step to remove the bacteria (9,000 × g, 2 min) the supernatants were stored at -20°C. Analysis of the cell supernatants for IL-8 protein secretion was performed using the BD OptEIA human IL-8 Kit (BD Biosciences, Heidelberg, Germany) according to the manufacturer's instructions.

For the NF-κB reporter assay, the stably transfected cell lines CaCo2, IPEC-J2 and HD-11, containing a NF-κB-Luc reporter fusion, were seeded at 2 × 10^4 ^cells in 200 μl medium per well in a 96 well plate and incubated for 24 hours. The medium was replaced before the addition of bacteria with the medium (100 μl per well) in the absence of antibiotics. The cell monolayers were infected with *C. jejuni *strains at an MOI of 50 in triplicates, centrifuged 5 min at 300 × g to sediment the bacteria, and coincubated for 1 h. After the coincubation, 50 μl of Steady-Glo^® ^Luciferase Assay Substrate (Promega Inc., Madison, WI, U.S.A.) was added to each well and incubated for 10 min to lyse bacteria and start the enzymatic reaction. The luminescence was then measured with a Wallac 1420 Victor3 V multilabel counter instrument (PerkinElmer, Waltham MA, U.S.A.).

### Generation of antisera

The *C. jejuni *antiserum 5699 was raised in rabbits against a mixture of six phylogenetically diverse strains, which were first fixed in paraformaldehyde, washed, and then administered to the animals by intradermal injection. The chicken antiserum M3 (generously provided by Bernd Kaspers) was obtained from a laying hen which was naturally infected with a *C. jejuni *strain (M3a, see Additional File 1: Table S1).

### Measurement of cellular ATP content

As an equivalent of intrabacterial energy harvest of each strain at specific culture conditions, intracellular ATP of different *C. jejuni *strains was measured. Bacteria were prepared as suspensions with O.D._600 _values of 0.3 (10^8 ^cells/ml) in RPMI 1640 defined cell culture medium at pH 7.4 (RPMI 1640 medium buffered with 20 mM HEPES; Invitrogen Inc., Darmstadt, Germany) Cells were taken directly from freshly grown plates (24 h growth, at 37°C) and resuspended gently in liquid medium without prior washing. The bacteria were then incubated for 30 min at 37°C before starting the ATP assay. For determining ATP contents of the cells, BacTiter-Glo reagent (an ATP-dependent luciferase-luciferin reagent mixture; Promega Inc., Madison, WI) was added directly to the live bacterial suspensions at a ratio of 1:1 after the incubation time. The suspensions were incubated at 37°C for 5 min to lyse the bacteria and to initiate the enzymatic reaction. The emission of photons was then measured with a Wallac 1420 Victor3 V multilabel counter instrument (PerkinElmer) in luminescence mode. All experiments were performed at least two times on separate days in duplicate measurements. Values were collected and further processed in MS Excel, calculating the level of luminescence (as relative luminescence units). In the results, one representative experiment is shown.

### DNA and protein methods

Genomic DNA of bacteria was extracted using the QIAamp DNA purification kit (Qiagen) according to the manufacturer's instructions. PCRs were performed with standard methods using Taq polymerase (Roche, Basel, Switzerland). PCR products prepared for Sanger sequencing were purified using the PCR purification kit (Qiagen, Hilden, Germany) according to the manufacturer's instructions before sequencing.

For the preparation of bacterial lysates, bacteria were grown for 24 h at 37°C or 42°C in a microaerobic atmosphere, harvested with sterile cotton swabs and resuspended in ice-cold 0.9% NaCl. The bacterial cells were sonicated three times 1 min with an ultrasonic device (Branson Sonic Power Company, Danbury, U.S.A.) at 4°C and at power level 5 (80-100 W). The amount of proteins in the lysates was analyzed using the BCA Protein Assay (Pierce, Rockford IL, USA). The proteins were separated on 11.5% SDS-polyacrylamide gels. Western immunoblotting was performed according to standard methods using the chicken antiserum M3 or our specific customary *C. jejuni*-mix antiserum 5699. Immuno-reactive bands were visualized with Super Signal West Pico chemiluminescent substrate (Pierce, Thermo Scientific, Bonn, Germany) on ECL hyperfilm (GE Healthcare, Piscataway NJ, U.S.A.).

### Molecular typing and pathotyping methods

492 *C. jejuni and C. coli *strains from our collection (473 of which were isolates collected between 2006 and 2010, the others were collected earlier and included some reference strains) comprising strains from different sources in Germany were MLST-typed as described in Dingle et al., 2005 [25]. Briefly, genomic DNA was amplified with primer pairs for seven housekeeping genes, *aspA, glnA, gltA, glyA, pgmA, tkt*, and *uncA*. The PCR-products were purified as described above and sequenced in both orientations using the Sanger method. The MLST data were collected in Seqsphere (RIDOM GmbH, Wuerzburg, Germany; http://www.ridom.de/seqsphere/) and Bionumerics (Applied Maths, Sint Martens-Latem, Belgium). Phylogenetic relationships between the STs were calculated by MLST-based cluster analysis in Bionumerics. Minimal Spanning Trees were generated from the cluster analysis with 100 permutations (Bionumerics), using the seven MLST loci information.

Based on the MLST analysis, 91 *C. jejuni *strains with representative STs were chosen for the PCR-based pathotyping. The presence or absence of selected putative virulence genes was detected by PCR as described above and in the results. For some genes with previously described sequence diversity, such as *ggt *and *ansB*, multiple primer combinations were tested to exclude false-negative results and to ensure that the absence of the gene is not due to the sequence diversity. The genes and primers are listed in Table 2.

**Table 2:** Oligonucleotide primers used for the amplification of *C. jejuni *genes

Gene	Primers	Sequence (5'-3')	**T**_**m**_^**a**^	Reference
*Cj0977*	Cj0977_F1 Cj0977_R1	TGAAGCACCCAAAAGAGTTTATATACG TTTCTGGATGTTTTGTAAATGAAAG	59	This study

*fspA1*	CjfspA1_F1 CjfspA1_R1	ATGTCGATTTAGTGTAATATATAT TAATCAGGCTTGGTGGCTTTGG	53	This study

*fspA2*	CjfspA2_F1 CjfspA2-RT04	AAACCGATAACAATATAAATTAATTTAAAAAG AAGCCTCTAATGCGGGATTT	53	This study [33]

*CjflaA*	Fla4F Fla1728R	GGATTTCGTATTAACACAAATGGTGC CTGTAGTAATCTTAAAACATTTTG	55	[63]

*FlaC*	CjflaC_F1 CjflaC_R1	CTAATACCATAATTGAACTCC TTCTACTATACCTTGATCAAAAAG	51	This study

*neuC1*	CjneuC1_F1 CjneuC1_R1	GGTGATAGAGTGGAGCCTTTAGCTG GTCAGTTCTACCATCTTGTCTTGAACC	49	[32]

*cadF*	CjcadF_F2B CjcadF_R1B	TTGAAGGTAATTTAGATATG CTAATACCTAAAGTTGAAAC	45	[64]

*cdtB*	CjcdtB_F CjcdtB_R	GTTGGCACTTGGAATTTGCAAGGC GTTAAAATCCCCTGCTATCAACCA	60	[65]

*cgtB*	DL39 cgtBrev	TTAAGAGCAAGATATGAAGGTG GCACATAGAGAACGCTACAA	53	[66]

*ciaB*	CjCiaB-F CjCiaB-R	CTATGCTAGCCATACTTAGGC GCCCGCCTTAGAACTTAC	51	[67]

*pglB*	pglBf pglBr	ACTTGGTGGGATTATGGTTATCCTG TCACGAGTTTTTGGTGTATCGATTTT	60	[68]

*ggt*	IF50 IF100	GGG TAA ATA AGA AGT TAG AAT TC CTT GAT AAA GGC GGA AAT GCC	55	[69]

*ggt*	Cjggt_fw1 Cjggt_rv1	TTTTAGCCATATCCGCTGCT AGCTGGAGTACCAGGAA	49	[12]

*ggt*	Cjggt_fw2 Cjggt_rv1	AAATAGCTTGGTATTGTGCT AGCTGGAGTACCAGGAA	49	This study

*ansB perip*.^b^	ansB_fw3_deg ansB_rv1	TWTYTATAGGAGYATGTA CAA ATA AAG CTT TTG CAG C	51	This study

*ansB* *secr*.^b^	ansB_fw2 ansB_rv1	CAA AAT AAG GGG AGA ATT GTG CAA ATA AAG CTT TTG CAG C	49	This study

*flpA*	CjflpA_fw1 CjflpA_rv1	CTGGTACTAAGTATCGTTATA AAGGAAGCTTGAGCTCGTA	51	This study

*tlp7*	tlp7-F01 tlp7-R01	AGGTTTCTGCTGCAATTTTTGTGGTG AGCAAGTTCTCCAAGTTCATTGCCA	60	[18]

*dmsA*	dmsA_F dmsA_R	GATAGGGCATTGCGATGAGT CTTGCTAGCCCAATCAGGAG	55	[12]

*Cj1585*	Cj1585c_F Cj1585c_R	TGTTGTGGGTTTGCTGGATA TTGCTTCACTGCATTCATCC	53	[12]

*capA*	CjcapA_F CjcapA_R	TGAATCGAAGTGGAAAAATAGAAG CCCATTTTTGTATCTTCATAACCT	60	[70]

*fucP*	CjfucP_F1 CjfucP_R1	CTTCATATTAGCCAGCATGA CCATATTAGCTACCAATGCA	51	This study

^a ^annealing temperature in °Celsius;^b ^perip. = periplasmic; secr. = secreted

### Metabolic phenotyping using Biolog Phenotype Microarrays

The bacteria were harvested from fresh plates after 24 h incubation at 37°C or 42°C, resuspended in IF-0a medium (Biolog, Hayward, CA, U.S.A.) and adjusted to 16% transmittance (O.D._600 _= 0.8). The cell suspension was mixed with a solution containing IF-0a, tetrazolium violet (Redox dye D), PM 1 additive and water according to the manufacturer's instructions (Biolog). 100 μl of the final suspension was added to each well of the 96-well Biolog carbon utilization plate PM1. The PM1 panels were placed without lids into gas impermeable bags (Biolog) together with a Campy Gen Compact sachet (Oxoid) to generate a microaerobic atmosphere and sealed. All working steps were carried out under the laminar flow to ensure a sterile environment. The Biolog panels were incubated in the OmniLog instrument (Biolog) either at 37°C or 42°C for 30 h. The utilization of different metabolites was recorded spectrophotometrically every 15 min over the whole incubation period, and evaluated with the OmniLog PM software (Kinetic Analysis). The area values (area under the kinetic curves) over time were used for comparison of substrate utilization between strains. All assays were repeated for at least three times on different days. Eight strains of ST-21 (xy259, A588, RB922, RB923, 6399, 7731, 04197, 04199) and nine, exclusively human-isolated, strains from other STs (xy898 [ST-50], R267 [ST-48], xy904 [ST-45], A396 [ST-267], 222 [ST-257], 02551 [ST-607], 02557 [ST-290], A412 [ST-51], A599 [ST-572]) were analyzed. Reproducibility of the assays was high, indicated by a low standard deviation of approximately 31%, calculated for three independent biological replicate assays (evaluated only for constantly positive area values > 800, since area values below 800 were evaluated not to be reliably positive). False-positive results for four pentose sugars, D-xylose, L-arabinose, D-ribose and L-lyxose, which were due to the sensitivity of the redox dye alone, as confirmed by incubating Biolog plates in the absence of bacteria, were excluded from the analysis. These false positive results have also been described before by others [59]. No other false positive results for saccharides were noted.

### *Campylobacter *whole genome sequencing and sequence analysis

Whole genomic DNA for 454 sequencing was isolated using Qiagen Genomic tip 100/G columns and the Genomic DNA Buffer Set (Qiagen). High quality genomic DNA of *C. jejuni *was then sheared according to a reproducible method by nebulization, quality-controlled on an Agilent bioanalyzer chip (DNA Chip7500, Agilent, Santa Clara, CA, U.S.A.), end-polished with T4 DNA polymerase and T4 polynucleotide kinase (New England Biolabs, Beverly MA, U.S.A.) [47]. DNA fragments were then subjected to bead coating and 454 sequencing in a Roche 454 sequencer, using FLX Titanium (Roche) chemistry according to the manufacturer's instructions.

Subsequent to the pyrosequencing process, the Roche Assembler software was used for assembling the primary reads into contigs. Between 37 and 156 contigs were obtained for the five whole genome sequences (xy259: 68 contigs, estimated genome size: 1,7 Mbp; RB922; 156 contigs, estimated genome size 1.7 Mbp; 6399: 43 contigs, estimated genome size 1.5 Mbp; 04197: 83 contigs, estimated genome size 1.7 Mbp; 04199: 71 contigs, estimated genome size 1.8 Mbp). Further statistics for the sequencing process and additional quality controls are available upon request. The draft genome sequences are available in the ENA EMBL-Bank database with accession numbers CAFR01000001-68, CAFS01000001-156, CAFT01000001-43, CAFU01000001-83, CAFV01000001-71. Further, the genomes were annotated in the Kodon software (Applied Maths, Sint Martens-Latem, Belgium) using several publicly available complete *C. jejuni *genome sequences, and the contigs of the draft sequences were then ordered 1) by aligning all five draft sequences to each other, and 2) by aligning all five genome sequences using the finished genome of *C. jejuni *11168 [20] as a scaffold. Whole genome alignments were generated within the Kodon, GeneDoc (http://www.psc.edu/biomed/genedoc) and MAUVE [60] softwares. All pairs of genomes (reference genome was 04199 for all pairwise comparisons) were analyzed using the Hidden Markov Model implemented in ClonalFrame [61] which comprises two states: the "clonal" state in which only mutations separate the two genomes and the "recombined" state in which the polymorphism density is higher due to the import of unrelated DNA. This allowed to pinpoint the regions that had recombined, as well as to quantify the overall frequency and effect of recombination during the diversification of pairs of genomes. A similar approach was recently implemented by Kennemann et al [47].

### Chicken infection

To evaluate the colonization ability of different *C. jejuni *strains, we infected three-weeks old specific-pathogen-free (SPF) white leghorn chicks (Lohmann Animal Health, Cuxhaven, Germany). Chicks were reared in cages and fed with a commercial diet devoid of antibiotics and provided with sterile drinking water ad libitum. The chicks (five animals per group) were inoculated by intraoesophagal gavage, twice on two subsequent days, each time with a 0.5 ml suspension containing 5 × 10^7 ^cfu/ml of a *C. jejuni *human- (xy259) or chicken-derived (RB922) strain, resuspended in Brain Heart Infusion (BHI, Oxoid) broth. One day prior to the infection, cloacal swabs were taken to confirm by plating and by genus- and species-specific PCR that the chickens were *Campylobacter*-free. On days 2, 3, 6, 9 and 13 after the second inoculation, cloacal swabs were again taken for continuous monitoring of the colonization levels. The swabs were first streaked onto Columbia blood agar plates supplemented with selective antibiotics (see above) and after that used for the extraction of chromosomal DNA according to the QiaAmp Tissue DNA spin kit protocol. The presence of *C. jejuni *strains in the infected animals was confirmed for the swab DNAs and the cultured bacteria by PCR with specific *Campylobacter *genus primers, based on the 16S ribosomal RNA gene sequence [62], and by primers specific for several *C. jejuni *genes. On day 15 after the second inoculation, the chickens were sacrificed. Caeca were sampled from all animals for DNA and RNA isolation. DNA was prepared from equivalent pieces of the caecal tip tissue for all animals using the QiaAmp Tissue DNA Spin kit (Qiagen). Colonization levels in the caecum were quantitatively evaluated for all animals using Real Time PCR on equal amounts of DNA prepared from caecal total tissue. All animal experiments were performed according to German legal requirements with approval by the federal government of Berlin permit from SoGeLa no. G 0210/09.

### RNA preparation

An inoculum of the *Campylobacter *strains was prepared from bacteria grown for less than 24 h on Columbia blood agar plates. The bacteria were inoculated at an initial O.D._600 _of approximately 0.08 into liquid medium (BHI Broth) in the presence of 5% horse serum (Oxoid). Bacteria were grown in liquid medium overnight, up to an O.D._600 _of 0.4 to 0.6. Bacteria were then harvested by centrifugation (21.000 × g for 2 min, room temperature), bacterial pellets flash frozen in liquid nitrogen and stored at -80°C. For RNA preparation, bacterial pellets were resuspended in Qiagen RNeasy lysis buffer containing b-mercaptoethanol and lysed in a beadbeater machine (Fastprep, Bio101 Thermo Scientific, Bonn, Germany) for 45 s. The RNA was further purified from the lysates using the Qiagen RNeasy protocol. Total RNA was quantitated in a Nanodrop 1000 device (Thermo Scientific, Bonn, Germany) and equal amounts of each sample subjected to DNAse I treatment (Roche). Residual content of DNA was determined by PCR using *Campylobacter *genus-specific 16S rDNA primers (Table 2), and, if necessary, the residual DNA was removed by a second DNAseI treatment and subsequent RNeasy column purification. cDNA was prepared from equivalent amounts (1 μg) of total RNA using Superscript III reverse transcriptase (Invitrogen Inc.) and using random hexamer primers according to the manufacturer's instructions. PCR or RT PCR using gene-specific primers (Table 2) was then performed on equal amounts of cDNA (0.5 to 2.5 μl) and normalized to a 16S rDNA RT PCR control reaction.

### Real time PCR (TaqMan)

The RT-PCR was performed using SYBR Green (Qiagen). Reactions were carried out on a Bio-Rad cycler (Abi TaqMan 7000 [ABI Applied Biosystems by Life Technologies, Darmstadt, Germany] and BioRad C1000/CFX96 combined system [BioRad, Hercules, CA, U.S.A:]). Primers targeted to the *pglB *gene (Table 2) were used to amplify sequences specific for *C. jejuni*. Cycling conditions were as follows: denaturation for 10 min at 95°C, amplification for 40 cycles at 95°C for 30 s, 60°C for 15 s, and at 72°C for 30 s. For quantification of DNA amounts, a serial standard was prepared from a purified PCR product of the *C. jejuni pglB *gene (strain xy259). Final concentrations of the standard were 10 pg, 1 pg, 0.1 pg, 0.01 pg and 0.001 pg of DNA. All standard probes were also prepared in a second serial dilution mixed with mouse caecum DNA at a final concentration of 100 ng per μl in each sample (spiked samples). The same concentration of total DNA was used in the final reactions for all DNAs prepared from infected chicken caeca. Each reaction was performed in duplicate.

## Authors' contributions

DHl, FK, BB, SW, SM, TJ, and PB performed experiments, KS, DS, MU and DHo contributed to methodology and data analysis, XD analyzed and discussed data, statistics and methodology, PG, GG, TA, TJ SM, LW LE, and KT provided materials, contributed to strain collections, experimental design and animal experiments, EG, SS, CJ designed and performed experiments, analyzed the data and wrote the paper. All authors read and approved the final manuscript.

## Supplementary Material

Additional file 1 **Suplementary Table S1**: all *Campylobacter *strains used in this study and MLST results. Click here for file

Additional file 2 **Supplementary Tables 2 to 9 and Supplementary Figures 1 to 4**. Supplementary Tables: Table S2-Pathotyping scheme of 91 *C. jejuni *strains ordered according to isolation source; Figure 2 in manuscript shows a subset of the 91 strains typed for an extended set of genes. Table S3-Carbon source utilization by eight ST-21 strains from different sources and nine human-derived strains of different STs; extended Biolog table, containing additional information on the Biolog PM1 substrates and quantitative area values Table S4-Whole genome sequence statistics of the five ST-21 strains. Table S5-Presence of unique or non-ubiquitous genes in the five whole genome sequences of ST-21 strains. Table S6-List of genes showing evidence of clustered recombination events in at least one of the five completely sequenced ST-21 genomes. Table S7-Variability between ST-21 *C. jejuni *strains in nucleotide repeats in phase-variable genes of flagellar glycosylation loci. Table S8-List of nucleotide variation and polymorphisms in intergenic regions in the five *C. jejuni *ST-21 genome sequences. Table S9-Distribution of selected phage-related genes within ST-21 strains from different sources. Supplementary Figures: Figure S1-Minimal spanning tree of all FBI-Zoo *Campylobacter *isolates colour-coded according to their assignment to the two *Campylobacter *species *C. jejuni *and *C. coli*. Figure S2-Minimal spanning tree of all FBI-Zoo *Campylobacter *isolates in the context of the PubMLST database (http://PubMLST.org). Figure S3-Gene categories (COG) in recombined stretches of sequence in ST-21 *C. jejuni *genome sequences. Genes in recombined stretches of the sequence of five ST-21 strains were grouped into COG categories indicating functional assignments; the graphic representation shows that no functional COG category is significantly overrepresented in the recombined gene clusters. Figure S4-Kodon snapshot from the alignment of five ST-21 strains with 04199 as reference; examples of microdiversity and clustered polymorphisms in a stretch of sequence compared between the complete genome sequences of five ST-21 *C. jejuni *strains are depicted. Click here for file

Additional file 3 **Supplementary Table S10**: Statistical analysis of *Campylobacter jejuni *ST-21 genome comparisons using a Bayesian statistical model; comparative statistical values for the proportion of clonal sites, where mutations or recombination were found in each strain, are indicated; average length of recombined regions and amount of polymorphism brought in by recombination in each strain are also listed. Click here for file
