# Supplementary material for: Closely related Campylobacter jejuni strains from different sources reveal a generalist rather than a specialist lifestyle
Source: BMC Genomics. 2011 Nov 28;12:584. doi: 10.1186/1471-2164-12-584 (PMC3283744; doi:10.1186/1471-2164-12-584)
Supplement: Additional file 2 — Supplementary Tables 2 to 9 and Supplementary Figures 1 to 4. Supplementary Tables: Table S2-Pathotyping scheme of 91 C. jejuni strains ordered according to isolation source; Figure 2 in manuscript shows a subset of the 91 strains typed for an extended set of genes. Table S3-Carbon source utilization by eight ST-21 strains from different sources and nine human-derived strains of different STs; extended Biolog table, containing additional information on the Biolog PM1 substrates and quantitative area values Table S4-Whole genome sequence statistics of the five ST-21 strains. Table S5-Presence of unique or non-ubiquitous genes in the five whole genome sequences of ST-21 strains. Table S6-List of genes showing evidence of clustered recombination events in at least one of the five completely sequenced ST-21 genomes. Table S7-Variability between ST-21 C. jejuni strains in nucleotide repeats in phase-variable genes of flagellar glycosylation loci. Table S8-List of nucleotide variation and polymorphisms in intergenic regions in the five C. jejuni ST-21 genome sequences. Table S9-Distribution of selected phage-related genes within ST-21 strains from different sources. Supplementary Figures: Figure S1-Minimal spanning tree of all FBI-Zoo Campylobacter isolates colour-coded according to their assignment to the two Campylobacter species C. jejuni and C. coli. Figure S2-Minimal spanning tree of all FBI-Zoo Campylobacter isolates in the context of the PubMLST database (http://PubMLST.org). Figure S3-Gene categories (COG) in recombined stretches of sequence in ST-21 C. jejuni genome sequences. Genes in recombined stretches of the sequence of five ST-21 strains were grouped into COG categories indicating functional assignments; the graphic representation shows that no functional COG category is significantly overrepresented in the recombined gene clusters. Figure S4-Kodon snapshot from the alignment of five ST-21 strains with 04199 as reference; examples of microdiversity and clus [file 1471-2164-12-584-S2.PDF]

strains	groups/STs	sources	Cj0977	CjFspA1	CjFspA2	CJFlaC	CjFla4	CjneuC1	CjCadF	CjcdtB	CjCiaB	CjpglB	Cjggt	CjansB	CjansBs	CjflpA	Cjtlp7	Cjdtlp7	Cj1585	CjdmsA
A 426	ST-464	human			*	*	*	*								*				
xy 897	gr 5			*		*	*							*						
xy 898	gr 7				*	*	*	*												
xy 249	ST-5388					*														
xy 248	ST-89		*	*		*	*	*		*	*	*				*				
xy 247	ST-831						*													
A 319	gr 7						*													
A 412	gr 8						*													
xy 259	gr 1																			
xy 358	ST-597				*	*	*	*												
xy 707	ST-5389				*	*	*	*												
xy 713	ST-1519		*		*	*	*	*	*	*	*	*				*				
xy 791	ST-658				*	*	*													
xy 901					*	*	*													
Typ 57	ST-403		*		*	*	*	*	*	*	*	*								
A 588	gr 1					*	*	*								*				
Ring 267	gr 6			*		*	*													
xy 904	gr 5		*	*		*	*		*	*	*	*		*		*				
A 76	gr 9				*	*	*													
A 310	ST-53				*	*	*	*												
A 318					*	*	*	*												
A 396	gr 3			*		*	*	*												
A 599	gr 9				*	*	*													
A 659	ST-883				*	*	*	*												
02557	gr 4					*		*				*		*		*				
02562	gr 7					*		*				*				*				
02551	gr 10					*		*				*								
02559	ST-824					*		*				*								
02553	ST-917					*		*				*								
02556	ST-5387					*		*				*								
0222	gr 2																			
02563	ST-5213					*		*				*								
04410	gr 1																			
04293																				
04299																				
04438																				
04448																				
chicken 1A	ST-2807	chicken	*	*		*	*		*	*	*	*				*				
chicken 4	ST-4800		*		*	*	*	*	*	*	*	*				*				
chicken 5A				*		*	*	*												
chicken M3A	ST-457																			
04329	ST-534					*		*				*								
04331	gr 10							*												
04207	ST-4810																			
04334	ST-2192					*		*				*								
04335								*												
04336								*												
RB 1008	gr 2			*		*						*		*		*				
RB 259	ST-760				*			*												
RB 922	gr 1																			
RB 923				*										*						
RB 957	gr 7				*			*												
RB 959	ST-3130				*	*		*				*								
RB 960	gr 8			*																
04347	gr 4							*						*						
04337	gr 6																			
04200	ST-141	bovine						*								*				
04195	gr 7							*								*				
04197	gr 1																			
04199																				
4834	gr 5	poultry meat		*		*						*				*				
4896	ST-46				*			*								*				
4897	ST-5428				*			*												
7063	ST-5398					*						*								
7080	ST-5211					*		*												
7321	gr 3							*												
6672	gr 4							*						*						
6797	gr 6																			
6823	gr 7							*												
7338	gr 8																			
7040	gr 9																			
7255	gr 1																			
7345																				
6399	gr 1	milk																		
7731																				
7732																				
7928																				
6660																				
6661																				
6278																				
7239																				
005	ST-5408	swine						*				*				*				
6345	gr 5	budgie				*						*								
6225	gr 3	dove						*												
6917	gr 1	lamb																		
6184	ST-905	sawyer																		
6316	ST-5386	duck				*						*								
6812	gr 1																			
6348	gr 7	dog				*		*				*								
6323	gr 5					*						*								
7072	gr 9																			

Table S2.: Pathotyping scheme of 91 *C. jejuni* strains ordered according to isolation source. Different ST groups of frequent STs comprising strains from more than one source are highlighted using the same color for each group (in the left two columns). The distribution of putative virulence- and host-associated genes was tested by PCR. Gr 1 – ST-21, gr 2 – ST-257, gr 3 – ST-267, gr 4 – ST-290, gr 5 – ST-45, gr 6 – ST-48, gr 7 – ST-50, gr 8 – ST-51, gr 9 – ST-572 and gr 10 – ST-607. Color-coding of genes: green: gene present; red: gene not present; yellow: weak PCR product; white: not tested. Strains marked by asterisks were additionally sequenced by the Sanger method to identify nucleotide polymorphisms.

			ST-21								A412	xy898	R267	xy904	A396	0222	02551	02557	A599
			xy259 (h)	A588 (h)	RB922 (ch	RB923 (ch	6399 (f)	7731 (f)	04197 (b)	04199 (b)	ST-51	ST-50	ST-48	ST-45	ST-267	ST-257	ST-607	ST-290	ST-572
PM01	Wells	Test																	
PM01	A01	Negative Control	0	0	0	0	0	0	0	0	0	0	0	0	0	0	0	0	0
PM01	A03	N-Acetyl-D-Glucosamine	0	0	0	0	0	0	0	0	0	0	0	0	1	0	0	0	0
PM01	A04	D-Saccharic Acid	19	4	0	0	0	0	0	0	0	0	0	0	129	0	0	0	0
PM01	A06	D-Galactose	48	99	0	16	0	25	7	27	0	334	0	0	180	0	0	0	0
PM01	A09	D-Alanine	0	3	0	0	0	0	0	0	0	18	0	0	208	0	0	0	0
PM01	A10	D-Trehalose	0	115	0	17	0	0	0	58	0	27	0	0	726	0	0	0	0
PM01	A11	D-Mannose	171	1366	448	1186	449	1260	114	1632	197	1992	198	0	1907	172	299	302	0
PM01	B01	D-Serine	65	0	43	0	22	0	3	0	0	0	2	10	0	0	2	0	0
PM01	B02	D-Sorbitol	0	0	0	0	0	0	0	0	0	0	0	0	0	0	0	0	0
PM01	B03	Glycerol	0	0	0	0	100	0	0	0	0	0	0	0	129	0	139	0	0
PM01	B05	D-Glucuronic Acid	0	0	0	0	0	0	0	0	0	0	0	0	196	0	19	0	0
PM01	B06	D-Gluconic Acid	0	0	0	0	0	0	0	0	0	0	0	0	8	0	0	0	0
PM01	B07	D,L-a-Glycerol- Phosphate	0	0	0	0	0	0	0	0	0	0	0	0	0	0	0	0	0
PM01	B11	D-Mannitol	0	0	71	0	107	0	0	0	0	0	0	0	64	0	0	0	0
PM01	C01	D-Glucose-6-Phosphate	281	0	324	0	113	0	163	0	0	0	879	81	116	0	2	0	0
PM01	C02	D-Galactonic Acid-g-Lactone	0	0	0	0	0	0	0	0	0	0	0	0	0	0	0	0	0
PM01	C05	Tween 20	1681	819	682	694	2009	200	0	0	731	2863	833	4142	1551	1103	0	27	0
PM01	C06	L-Rhamnose	0	20	12	1	0	1	9	0	0	0	0	0	2	0	0	0	0
PM01	C07	D-Fructose	259	25	0	1	1	0	35	2	0	654	582	0	731	0	439	0	0
PM01	C08	Acetic Acid	0	0	0	0	469	0	0	0	0	11	1165	0	1026	0	255	0	0
PM01	C09	a-D-Glucose	0	0	0	0	0	0	0	0	0	0	0	0	60	0	0	0	0
PM01	C10	Maltose	0	0	0	0	0	0	0	0	0	0	0	0	14	0	0	0	0
PM01	C11	D-Melibiose	0	0	0	0	0	0	0	0	0	0	2	0	147	0	3	0	0
PM01	C12	Thymidine	105	1136	1533	840	1255	913	288	938	61	1361	15	213	1562	376	775	376	1
PM01	D02	D-Aspartic Acid	0	0	0	0	0	0	0	0	0	0	0	0	0	0	0	0	0
PM01	D03	D-Glucosaminic Acid	0	0	0	0	0	0	0	0	0	0	0	0	0	0	0	0	0
PM01	D04	1,2-Propanediol	0	0	0	0	0	0	0	0	0	0	0	0	0	0	0	0	0
PM01	D05	Tween 40	0	0	0	0	30	0	0	0	0	606	80	0	21	384	232	0	0
PM01	D07	a-Keto-Butyric Acid	1864	1	232	0	2596	5	0	0	0	2647	2132	3172	914	0	0	0	0
PM01	D08	a-Methyl-D-Galactoside	0	0	0	0	0	0	0	0	0	0	0	0	2	0	0	0	0
PM01	D09	a-D-Lactose	0	0	0	0	0	0	0	0	0	0	0	0	55	0	0	0	0
PM01	D10	Lactulose	0	0	0	0	0	0	0	0	0	0	0	0	250	0	101	0	0
PM01	D11	Sucrose	0	0	0	0	0	0	0	0	0	0	0	0	19	0	0	0	0
PM01	D12	Uridine	27	863	1134	631	782	855	144	871	0	863	1	88	1192	91	201	295	0
PM01	E03	D-Glucose-1-Phosphate	0	0	0	0	0	0	0	0	0	0	0	0	0	0	0	0	0
PM01	E05	Tween 80	0	0	0	0	5	0	0	0	0	44	0	0	11	0	0	0	0
PM01	E06	a-Hydroxy Glutaric Acid-g-Lactone	0	0	0	0	0	0	0	0	0	0	0	0	24	0	46	0	0
PM01	E08	b-Methyl-D-Glucoside	0	0	0	0	0	0	0	0	0	0	0	0	24	0	0	0	0
PM01	E09	Adonitol	0	0	0	0	0	0	0	0	0	0	0	0	0	0	0	0	0
PM01	E10	Maltotriose	0	0	0	0	0	0	0	0	0	0	0	0	110	0	0	0	0
PM01	E11	2`-Deoxy-Adenosine	0	0	0	0	0	0	1	0	0	0	0	0	0	0	0	0	0
PM01	F03	m-Inositol	0	0	0	0	0	0	0	0	0	0	0	2	0	0	0	0	0
PM01	F04	D-Threonine	0	0	0	0	0	0	0	0	0	0	0	0	0	0	0	0	0
PM01	F07	Propionic Acid	0	0	0	0	361	0	0	0	0	1459	0	0	683	0	0	0	0
PM01	F08	Mucic Acid	14	0	22	0	0	0	11	0	0	0	0	0	346	0	177	0	0
PM01	F11	D-Cellobiose	0	23	3	0	86	0	22	1	0	0	0	19	197	0	51	0	0
PM01	F12	Inosine	428	1019	1995	1311	1314	1552	754	1306	664	1927	59	555	1366	421	922	267	6
PM01	G02	Tricarballylic Acid	55	0	25	0	45	0	74	0	0	0	63	274	4	0	98	0	0
PM01	G04	L-Threonine	278	0	0	0	291	0	0	0	0	0	0	264	336	0	0	0	0
PM01	G05	L-Alanine	0	0	0	0	0	0	0	0	0	0	0	1	0	0	0	0	0
PM01	G06	L-Alanyl-Glycine	0	0	0	0	0	0	0	0	0	0	0	0	0	0	0	0	0
PM01	G07	Acetoacetic Acid	33	0	74	0	4	0	34	0	0	0	0	30	66	0	165	0	0
PM01	G08	N-Acetyl-b-D-Mannosamine	0	0	0	0	0	0	8	0	0	0	0	7	0	0	0	0	0
PM01	H09	L-Galactonic Acid-g-Lactone	684	0	1833	0	1732	0	978	0	0	0	113	1486	0	0	1757	0	0
PM01	E12	Adenosine	342	828	1422	1089	877	1412	238	1114	439	2449	579	226	927	67	106	231	0
PM01	A12	Dulcitol	910	4249	2189	3699	1876	4187	1134	4223	3050	5328	557	1130	4562	3211	2080	3555	201
PM01	H04	Tyramine	714	0	1215	0	1364	0	900	0	0	1	1163	754	4	0	1380	0	48
PM01	E04	D-Fructose-6-Phosphate	1443	1127	2160	1213	984	814	2365	1623	1112	589	558	972	173	100	253	0	371
PM01	H03	m-Hydroxy-Phenylacetic Acid	1845	0	1480	0	1729	0	1197	0	0	0	2121	1297	0	0	380	0	692
PM01	H07	Glucuronamide	3733	2041	4122	1737	4376	1615	3381	1811	518	3936	5434	3796	1902	281	3031	173	1574
PM01	H11	b-Phenylethylamine	3312	151	4659	234	3858	216	3403	288	0	1921	2939	3060	398	3	3216	0	305
PM01	H10	D-Galacturonic Acid	3314	514	4468	659	5043	676	3141	463	0	2672	2993	5226	1206	1976	5002	944	922
PM01	F10	Glyoxylic Acid	620	3096	4892	3204	1246	3633	2998	2827	506	481	3	861	3155	0	0	0	0
PM01	H02	p-Hydroxy-Phenylacetic Acid	3031	0	2401	0	3062	0	2193	0	0	64	2679	2887	30	0	634	0	2262
PM01	F09	Glycolic Acid	3551	4022	6453	4043	1940	4113	4316	3848	1520	1022	825	2586	5136	740	532	0	81
PM01	F01	Glycyl-L-Aspartic Acid	2694	0	2155	0	3467	81	1958	0	1094	569	4640	5207	1169	65	3462	0	4917
PM01	G01	Glycyl-L-Glutamic Acid	2420	0	2778	0	2622	0	2386	0	3072	0	2713	7594	5242	0	3080	0	4195
PM01	B10	Formic Acid	8079	4490	5899	6192	3512	5251	5244	7275	1168	2862	1822	4902	1930	0	1041	0	0
PM01	A08	L-Proline	4054	6156	6353	6261	3437	5410	3906	5743	2424	1446	0	1513	3842	509	0	198	51
PM01	A07	L-Aspartic Acid	7421	5762	7641	6840	2753	4355	6155	5738	3202	1186	264	3099	6697	3572	114	0	146
PM01	H12	Ethanolamine	4899	1098	6074	989	5911	1504	4957	1083	628	3700	5957	5556	1858	85	4228	6	1468
PM01	H01	Glycyl-L-Proline	4748	0	4688	0	5630	0	4719	0	1705	32	4914	6817	1330	32	5766	0	5801
PM01	E02	m-Tartaric Acid	1621	686	2871	194	845	751	3067	599	1359	743	13	1677	0	0	0	0	207
PM01	F05	Fumaric Acid	9280	4179	9240	5648	4041	2363	8692	4950	1383	106	1510	4740	5528	1000	0	0	292
PM01	G09	Mono Methyl Succinate	7230	5566	11047	7681	5889	6745	8504	7190	5038	3149	4979	8177	8061	4423	3051	197	5473
PM01	B12	L-Glutamic Acid	8350	8244	9055	7084	6980	8045	5031	6965	6992	3332	3374	6849	8358	4894	3390	3174	4217
PM01	D01	L-Asparagine	10014	5016	9491	5704	4320	4152	8216	4150	3799	409	2576	6251	5665	1480	1219	0	3623
PM01	E01	L-Glutamine	7894	2926	7713	3294	5896	2148	4158	2750	4021	0	2543	7993	6300	150	1889	11	3556
PM01	A05	Succinic Acid	10483	8954	11367	10167	5866	9610	8953	8660	8136	6508	7026	8686	10877	7515	4389	4181	8324
PM01	H05	D-Psicose	6810	5869	8775	5850	6970	4802	9112	6151	907	4856	5958	6244	3066	3830	6619	2016	3924
PM01	F06	Bromo-Succinic Acid	8979	5893	7550	5025	2128	4318	7101	3934	1721	1274	2715	3399	6742	915	0	0	229
PM01	G12	L-Malic Acid	12325	9647	13719	10521	7236	8390	12748	9619	6345	4667	4641	9100	8794	6418	2849	993	4021
PM01	B08 *	D-Xylose	9916	12406	12055	11271	9538	11320	11434	12953	9186	10928	9181	9489	10095	8380	6359	7888	8380
PM01	B04	L-Fucose	5051	4210	7061	6716	7815	4463	3737	7722	13668	9189	10981	0	245	8906	7468	2693	5778
PM01	G03	L-Serine	9887	5193	9914	5807	6251	6196	10708	6536	8269	755	0	7418	7022	0	0	3	0
PM01	D06	a-Keto-Glutaric Acid	13363	14055	15776	13445	10166	12603	15295	16463	11741	6929	9145	11269	11793	9665	2400	3955	9311
PM01	E07	a-Hydroxy-Butyric Acid	14336	13427	13447	9973	7739	10153	13793	11021	12030	9862	9116	12412	14787	9824	530	4061	8199
PM01	A02 *	L-Arabinose	11997	12907	13361	12027	11650	11836	12744	13188	11116	14012	13445	12659	9967	11963	9070	10036	13460
PM01	G10	Methyl Pyruvate	16687	13453	15908	12464	10927	8943	16665	14525	12882	10140	9634	12848	11251	8157	4945	3846	9478
PM01	F02	Citric Acid	10653	6749	11853	6911	7461	4927	11974	10947	7760	1098	2688	10377	7152	4172	788	0	6503
PM01	B09	L-Lactic Acid	17571	17531	17228	15858	12660	15662	16696	16836	16493	14211	13306	14104	18867	15705	11255	11459	13148
PM01	C03	D,L-Malic Acid	15474	9776	12643	8477	4997	6478	13016	7238	5819	3311	6689	8001	8721	5603	2773	0	4326
PM01	G11	D-Malic Acid	15323	13214	14960	10673	6371	12100	14348	10676	13501	8194	9171	10278	12789	8755	6254	798	5833
PM01	C04 *	D-Ribose	16065	15130	17110	14074	14300	13908	16828	15486	13094	14670	15353	14153	13724	10742	11482	9774	13332
PM01	H08	Pyruvic Acid	20839	17554	20259	17379	17276	16426	20739	18514	16323	15711	16641	17631	15516	13897	10427	10773	15912
PM01	H06 *	L-Lyxose	19140	17873	21621	17017	19697	16951	20854	18055	14721	17607	19452	19167	16779	13758	16838	12561	16158

Table S3: Carbon source utilization by eight ST-21 strains (left panel) from different sources (h = human; ch = chicken; f = food; b = bovine) and nine human-derived strains of different STs (right panel; strain designation see Table S1) were tested at 42°C using Biolog Phenotype Microarray PM1 plates (mean of area values at 30 h incubation, derived from three independent experiments are depicted for all ST-21 strains). Grey color indicates no or only weak substrate utilization (all area values below 800) for all strains and green means high substrate utilization (all area values above 800) for all strains. The colors pink, yellow, beige and blue indicate strain-specific utilization: pink – zero utilization, beige - < 1000, yellow - <9000, blue - >9000. The identities and localization of all metabolic substrates in 95 wells of the BioLog PM1 plate are shown on the left (columns 2 and 3). Substrates that are not utilized by ST-21 biotype 1 strains are indicated by boxed values. * indicate false positive values

Strain of ST21	genome size	no. of contigs	no. of reads	coverage
xy259 (h)	1.71 Mbp	68	132,478	≈ 35 fold
RB922 (ch)	1.71 Mbp	156	147,368	≈ 40 fold
6399 (f)	1.62 Mbp	43	141,239	≈ 42 fold
04197 (b)	1.7 Mbp	83	121,133	≈ 29 fold
04199 (b)	1.8 Mbp	71	133,896	≈ 31 fold

Table S4: Whole genome sequence statistics of the five ST-21 strains; h = human, ch = chicken, f = food, b = bovine

04197	04197 + RB922 phage cluster 1 (CJIE4)	04197 + 04199 + RB922 phage cluster 2 (CJIE1 in RM1221)	04197 + 04199 gene cluster 3 of unknown origin
**The follows genes are part of the phage 2, indicated by black arrows in the Fig 5C after the common phage gene DAM (in grey):**<br>2.4 tail protein D<br>2.5 phage tail protein<br>2.6 probable protein encoding sequence<br>2.7 probable protein encoding sequence<br>2.8 phage major tail tube protein	**04197: flanking genes *Cj1282* (*mrdA*) and *Cj1283* (*ktrB*)**<br>**RB922: flanking gene Cj1282 (mrdA)**<br>1.1 site-specific recombinase, phage integrase family (CJE1418)<br>1.2 hypothetical protein (CJE1420)<br>1.3 site-specific DNA methyltransferase (CJE1421)<br>1.4 hypothetical protein (CJE1423)<br>1.5 hypothetical protein (CJE1425)<br>1.6 hypothetical protein (CJE1427)<br>1.7 RloG protein (CJE1430)<br>1.8 DNA binding protein Roi (CJE1435)<br>1.9 hypothetical protein (CJE1439)<br>1.10 signal peptidase I (CJE1440)<br>1.11 DNA/RNA non-specific endonuclease (CJE1441)<br>1.12 hypothetical protein (CJE1443)<br>1.13 hypothetical protein (CJE1444)<br>1.14 hypothetical protein (CJE1445)<br>1.15 hypothetical protein (CJE1447)<br>1.16 hypothetical protein (CJE1448)<br>1.17 hypothetical protein (CJE1449) [RB922 only]<br>1.18 hypothetical protein (CJE1450)<br>1.19 hypothetical protein(CJE1451)<br>1.20 hypothetical protein (CJE1452)<br>1.21 hypothetical protein (CJE1453)<br>1.22 phage head-tail adaptor (CJE1454)<br>1.23 hypothetical protein (CJE1455)<br>1.24 hypothetical protein (CJE1457)<br>1.25 major capsid protein, HK97 family (CJE1458)<br>1.26 hypothetical protein (CJE1459)<br>1.27 hypothetical protein (CJE1460)<br>1.28 hypothetical protein (CJE1461)<br>1.29 conserved hypothetical protein (CJE1463)<br>1.30 hypothetical protein (CJE1465)<br>1.31 hypothetical protein (CJE1466)<br>1.32 hypothetical protein (CJE1467)<br>1.33 phage protein HK97 gp10 family (CJE1468)<br>1.34 portal protein HK97 family (CJE1469)<br>1.35 toxin-antitoxin protein (CJE1470) *<br>1.36 phage terminase, large subunit (CJE1471)<br>1.37 phage terminase, small subunit (CJE1472)<br>1.38 HNH endonuclease domain protein (CJE1473)	**04197: flanking genes *Cj1474* and *Cj1473***<br>**04199: insertion in *Cj1146***<br>**RB922: not identified**<br>2.1 phage repressor protein (CJE0215)<br>2.2 hypothetical protein (CJE0216)<br>2.3 DNA adenine methylase DAM (CJE0220)<br>2.4 phage virion morphogenesis protein (CJE0221), [04199,RB922]<br>2.5 tail tape measure protein TP901 family (CJE0222) [04199,RB922]<br>2.6 hypothetical protein (CJE0223) [04199, RB922]<br>2.7 conserved domain protein (CJE0225) [04199, RB922]<br>2.8 phage major tail tube protein (CJE0226) [04199, RB922]<br>2.9 major tail sheath protein (CJE0227)<br>2.10 hypothetical protein (CJE0228)<br>2.11 conserved hypothetical protein (CJE0229)<br>2.12 hypothetical protein (CJE0230)<br>2.13 tail fiber protein H (CJE0231)<br>2.14 phage tail protein (CJE0232)<br>2.15 baseplate assembly protein J (CJE0233)<br>2.16 baseplate assembly protein V (CJE0236)<br>2.17 conserved hypothetical protein (CJE0237)<br>2.18 conserved hypothetical protein (CJE0241)<br>2.19 hypothetical protein (CJE0243) [04199 + RB922)]<br>2.20 Mu-like prophage I protein (CJE0244) [04199 + RB922)]<br>2.21 hypothetical protein (CJE0245) [04199 + RB922]<br>2.22 conserved hypothetical protein (CJE0246) [04199 + RB922]<br>2.23 conserved domain protein (CJE0247) [04199 + RB922]<br>2.24 hypothetical protein (CJE0248) [04199 + RB922]<br>2.25 phage uncharacterized protein (CJE0249) [04199 + RB922]<br>2.26 hypothetical protein (CJE0250) [04199 + RB922]<br>2.27 prophage MuSo1, F protein (CJE0251) [04199 + RB922]<br>2.28 phage tail protein (CJE0252) [04199 + RB922]<br>2.29 tail protein D (CJE0254) [04199 + RB922]<br>2.30 extracellular deoxyribonuclease dns (CJE0256) [04199 + RB922]<br>2.31 conserved domain protein (CJE0258) [04199 + RB922]<br>2.32 hypothetical protein (CJE0259) [04199 + RB922]<br>2.33 hypothetical protein (CJE0261) [04199 + RB922]<br>2.34 conserved hypothetical protein TIGR01671 (CJE0262) [RB922 + 04197] *<br>2.35 host nuclease inhibitor protein Gam (CJE0265)<br>2.36 conserved hypothetical protein (CJE0266) [04199 + RB922]<br>2.37 bacteriophage DNA transposition protein B (CJE0269) [04199 + RB922]<br>2.38 bacteriophage DNA transposition protein A (CJE0270) [04199 + RB922]	3.1 probable protein encoding sequence<br>3.2 probable protein encoding sequence<br>3.3 probable protein encoding sequence<br>3.4 probable protein encoding sequence<br>3.5 probable protein encoding sequence<br>3.6 probable protein encoding sequence<br>3.7 probable protein encoding sequence<br>3.8 probable protein encoding sequence<br>3.9 probable protein encoding sequence<br>3.10 probable protein encoding sequence<br>3.11 probable protein encoding sequence<br>3.12 probable protein encoding sequence<br>3.13 Cpp32 (80% homology plasmid pTet)<br>3.14 probable protein encoding sequence<br>3.15 probable protein encoding sequence<br>3.16 probable protein encoding sequence<br>3.17 probable protein encoding sequence<br>3.18 probable protein encoding sequence<br>3.19 probable protein encoding sequence<br>3.20 probable protein encoding sequence<br>3.21 probable protein encoding sequence<br>3.22 probable protein encoding sequence

04199 unique phage cluster 4	xy259	
4.1 probable protein encoding sequence<br>4.2 probable protein encoding sequence<br>4.3 probable protein encoding sequence<br>4.4 probable protein encoding sequence<br>4.5 probable protein encoding sequence<br>4.6 probable protein encoding sequence<br>4.7 cpp32 (88% homology plasmid pTet)<br>4.8 probable protein encoding sequence<br>4.9 probable protein encoding sequence<br>4.10 probable protein encoding sequence<br>4.11 probable protein encoding sequence<br>4.12 probable protein encoding sequence<br>4.13 probable protein encoding sequence<br>4.14 probable protein encoding sequence<br>4.15 probable protein encoding sequence<br>4.16 probable protein encoding sequence	**Phage cluster 5**<br>**flanking genes *Cj1283* and *Cj1282* (*mrdA*)**<br>5.1 F-Box only protein *<br>5.2 hypothetical protein<br>5.3 putative sex pilus assembly<br>5.4 putative conjugative transfer protein TraE<br>5.5 hypothetical protein<br>5.6 sex pilus assembly protein<br>5.7 conserved hypothetical protein<br>5.8 putative lipoprotein<br>5.9 hypothetical protein<br>5.10 putative pilus assembly protein<br>5.11 putative type IV secretory protease<br>5.12 putative TraG<br>5.13 probable protein encoding sequence<br>5.14 probable protein encoding sequence<br>5.15 conserved hypothetical protein<br><br>**Phage cluster 6**<br>6.1 hypothetical protein<br>6.2 hypothetical protein<br>6.3 cpp32<br>6.4 hypothetical protein<br>6.5 hypothetical protein<br>6.6 DNA transfer in the process of con., F pilus assembly protein<br>6.7 hypothetical protein<br>6.8 lytic transglycosylase, catalytic<br>6.9 hdod domain superfamily<br>6.10 hypothetical protein<br>6.11 hypothetical protein<br>6.12 probable protein encoding sequence<br>6.13 probable protein encoding sequence<br>6.14 probable protein encoding sequence<br>6.15 probable protein encoding sequence<br>6.16 probable protein encoding sequence<br>6.17 probable protein encoding sequence	**Phage cluster 7 (homology to CJIE3)**<br>7.1 hypothetical protein (CJE1094, CJIE3)<br>7.2 probable protein encoding sequence<br>7.3 repeat motif-containing gene<br>7.4 hypothetical protein<br>7.5 hypothetical DNA primase<br>7.6 hypothetical protein<br>7.7 hypothetical protein<br>7.8 conserved hypothetical protein<br>7.9 probable protein encoding sequence<br>7.10 hypothetical protein<br>7.11 hypothetical protein<br>7.12 conserved hypothetical protein<br>7.13 probable protein encoding sequence<br>7.14 thymidine kinase<br>7.15 probable protein encoding sequence<br>7.16 probable protein encoding sequence<br>7.17 probable protein encoding sequence<br>7.18 probable protein encoding sequence<br>7.19 putative prophage LambdaCh01, recombination protein Bet<br>7.20 single-stand binding protein family<br>7.21 hypothetical protein<br>7.22 helix-turn-helix domain protein<br><br>**Phage cluster 8 (homology to CJIE3)**<br>8.1 TraG-like protein (CJE1107, CJIE3)<br>8.2 hypothetical protein<br>8.3 probable protein encoding sequence<br>8.4 probable protein encoding sequence<br>8.5 putative thioredoxin *<br>8.6 TraT complement resistence protein<br>8.7 hypothetical protein<br>8.8 hypothetical protein<br>8.9 putative protein binding<br>8.10 probable protein encoding sequence<br>8.11 hypothetical cytosolic protein<br>8.12 hypothetical protein<br>8.13 hypothetical protein<br>8.14 probable protein encoding sequence<br>8.15 hypothetical protein<br>8.16 conserved hypothetical protein TIGR01671 (04197, RB922) *<br>8.17 hypothetical protein<br>8.18 hypothetical protein<br>8.19 hypothetical protein<br>8.20 probable protein encoding sequence<br>8.21 probable protein encoding sequence<br>8.22 DNA/RNA non-specific endonuclease (xy259 + 04197 + RB922)

Table S5: Presence of unique or non-ubiquitous genes in the five whole genome sequences of ST-21 strains 04197 (b), 04199 (b), RB922 (ch) and xy259 (h); the genome of 6399, isolated from milk, has no unique genes. Most genes in these categories were identified to be phage-related genes, and some of them overlapped between strains as indicated on top of the table columns. Phage gene cluster 2, which is partially common between strains 04197, 04199 and RB922, is also depicted graphically in Fig 5C. All genes in each of the clusters are numbered in the order of appearance in the genomes. For some phage gene clusters, no flanking genes are listed, as the identification of flanking genes was not possible because of contig ends. The numbering of the flanking genes corresponds to the gene names in the genome of reference strain *C. jejuni* 11168 (Parkhill et al., 2000). Phage insertions and single genes with homology to *Campylobacter jejuni*-integrated elements (CJIEs; CJIE1, CJIE3, CJIE4) genes in strain RM1221 (Fouts et al., 2005) are indicated by the corresponding gene number of the genome of RM1221 and in the respective column headers. Presence of genes marked with asterisks was tested among other ST-21 strains, see Table S9.

	Cluster	COG	xy259 (h)	RB922 (ch)	6399 (f)	04197 (b)	04199 (b)
cluster *Cj0001-0029*	*Cj0001* , *dnaA* , chromosomal replication initiator protein	L					
	Cj0002, dnaN , DNA-Polymerase III, beta chain	L					
	Cj0003, gyrB, DNA gyrase subunit B	L					
	Cj0004, monohaem cytochrome C	-					
	Cj0005 , molydopterin containing oxidoreductase	R					
	Cj0006 , putative Na+/H+ antiporter family protein	R					
	Cj0007, gltB , glutamate synthase subunit	E					
	Cj0008 , conserved hypothetical protein	S				absent	
	Cj0009 , *gltD* , glutamate synthase small subunit	ER					
	Cj0010 , *rnhB* , ribonuclease HII	L					
	Cj0012 , *rrc* , non-haem iron protein	C					
	Cj0013, ilvD , dihydroxy-acid dehydratase	EG					
	Cj0014, putative integral membrane protein	S	del of 128 bp				del of 128 bp
	Cj0015 , hypothetical protein	U					
	Cj0016 , putative transcriptional regulatory protein	R					
	Cj0017 , *dsb* I, disulphide bond formatin protein	O					
	Cj0019 , putative MCP-domain signal transduction protein	NT					
	Cj0020 , *ccpA-1* , cytochrome C551 peroxidase	P					
	Cj0021 , putative fumarylacetoacetate (FAA) hydrolase family protein	Q					
	Cj0022 , RNA pseudouridylate synthase family protein	J					
	Cj0023 , *purB* , adenylosuccinate lyase	F					
	Cj0024 , *nrdA,* ribonucleoside-diphosphate reductase alpha chain	F					
	Cj0025 , sodium/dicarboxylate symporter	R					
	Cj0026 , *thyX* , thymidylate synthase	F					
	Cj0027 , *pyrG* , CTP synthetase	F					
	Cj0028 , *recJ* , putative single-stranded-DNA-specific exonuclease	L					
	Cj0029 , *ansB* , cytoplasmic L-asparaginase	EJ					
cluster *Cj0034-0044*	*Cj0034* , periplasmic protein	S					
	Cj0035 , putative efflux protein	G					
	Cj0036 , hypothetical protein	S					
	Cj0037 , putative cytochrome C	C					
	Cj0038 , hypothetical protein	-					
	Cj0039 , *typA* , GTP-binding protein	T					
	Cj0040, hypothetical protein	-					
	Cj0041 , *fliK* , putative flagellar hook-length control protein	-					
	Cj0042 , *flgD* , putative flagellar hook assembly protein	N					
	Cj0043 , *flgE* , flagellar hook protein	N					
	Cj0044 , hypothetical protein	-					
cluster *Cj0089-0091*	*Cj0088* , *dcuA* , anaerobic C4-dicarboxylate transporter	R					
	Cj0089 , putative lipoprotein	S					
	Cj0090 , putative lipoprotein	R					
	Cj0091 , putative lipoprotein	R					
cluster *Cj0116-0117*	*Cj0116* , *fabD* , malonyl coa-acyl carrier protein transacylase	I					
	Cj0117 , *pfs* , 5-methyltioadenosine/S-adenosylhomocysteine nucleosidase	F					
cluster *Cj0140-0142*	*Cj0140* , hypothetical protein	V					
	Cj0141 , putative ABC transporter integral membrane protein	P					
	Cj0142 , putative ABC transporter ATP-binding protein	P					
	Cj0143 , putative ABC transporter ATP-binding protein	P					
cluster *Cj0262-0264*	*Cj0262* , methyl-accepting chemotaxis protein	NT					
	Cj0263 , *zupT* , zinc transporter	P					
	Cj0264 , molybdopterin containing oxidoreductase	C					
cluster *Cj0274-0276*	*Cj0274, lpxA* , acyl-UDP-N-acetylglucosamine O-acyltransferase	M					
	Cj0275 , *clpX* , ATP-dependent Clp protease	O					
	Cj0276 , *mreB* , homolog of E.coli rod-shape protein	D					
cluster *Cj0349-0353*	*Cj0349* , *trpA* , tryptophan synthase alpha chain	E					
	Cj0350 , hypothetical protein	-					
	Cj0351 , *fliN* , flagellar motor switch protein	NU					
	Cj0352 , putative transmembrane protein	-					
	Cj0353 , phosphatase	FP					
cluster *Cj0365-0367*	*Cj0365* , *cmeC* , outer membrane channel protein	MU					
	Cj0366 , *cmeB* , inner membrane efflux transporter	V					
	Cj0367 , *cmeA* , periplasmic fusion protein	M					
cluster *Cj0386-0390*	*Cj0386* , *engA* , putative GTP-binding protein	R					
	Cj0387 , *aroK* , shikimate kinase	E					
	Cj0388 , *trpS* , tryptophanyl-tRNA-synthetase	J					
	Cj0389 , *serS* , seryl-tRNA synthetase	J					
	Cj0390, putative transmembrane protein	-					

Table S6 (including the following 3 pages): List of genes showing evidence of clustered recombination events in at least one of the five completely sequenced ST-21 genomes. All gene clusters showing recombination were extracted from rounds of multiple alignments of the five genomes in the Kodon software (Applied Maths, Sint Martens-Latem, Belgium), where, in each round, each of the five genomes was used in turn as a reference for the others in order to detect all recombination events. Empty cells indicate identical sequences between more than one strain. The genes showing evidence of recombination in each single genome are indicated by hatched cells. Some recombination events overlap between some strains and can consist of unrelated polymorphisms in others (not indicated).

	Cluster	COG	xy259 (h)	RB922 (ch)	6399 (f)	04197 (b)	04199 (b)
cluster *Cj0434-0438*	*Cj0434* , *pgm* , 2,3-bisphosphoglycerate-ind. phosphoglycerate mutase	G					
	Cj0435 , *fabG* , 3-oxoacyl(acyl-carrier-protein) reductase	IQR					
	Cj0436 , putative pyridoxamine 5´-phosphate oxidase	S					
	Cj0437 , *sdhA* , succinate dehydrogenase	C					
	Cj0438 , *sdhB* , putative succinate dehydrogenase iron-sulfur protein	C					
cluster *Cj0495-0497*	*Cj0495* , putative methyltransferase domain protein	R					
	Cj0496 , conserved hypothetical protein	R					
	Cj0497 , putative lipoprotein	-					
cluster *Cj0599-0605*	*Cj0599* , putative OmpA family membrane protein	M					
	Cj0600 , hypothetical protein	E					
	Cj0601, putative sodium-dependent transmembrane transport protein	R					
	Cj0602 , MOSC-domain containing protein	S					
	Cj0603 , *dsbD* , putative thiol:disulphide interchange protein	OC					
	Cj0604 , putative polyphosphate kinase	S					
	Cj0605 , putative amidohydrolase	R					
cluster *Cj0613-0614*	*Cj0613, pstS* , putative periplasmic binding protein	P					
	Cj0614 , *pstC* , putative phosphate transport system permeas protein	P					
	Cj0615 , *pstA* , putative phosphate transport system permeas protein	P					
cluster *Cj0619-0637*	*Cj0619* , MATE efflux family protein	V					
	Cj0620 , conserved hypothetical protein	R					
	Cj0621 , hypothetical protein	-					
	Cj0622 , *hypF* , carbamoyltransferase	O					
	Cj0623 , *hypB* , hydrogenase accessory protein	OK					
	Cj0625 , *hypD* , hydrogenase isoenzymes formation protein	O					
	Cj0626 , *hypE* , hydrogenase isoenzymes formation protein	O					
	Cj0627 , *hypA* , hydrogenase expression/formation protein	R					
	Cj0628, putative lipoprotein	-					
	Cj0630 , *holA* , DNA-Polymerase III, delta subunit	L					
	Cj0631 , putative ribonuclease	K					
	Cj0632 , *ilvC* , keto-acid reductoisomerase	EH					
	Cj0633, periplasmic protein	S					
	Cj0634 , *dprA* , DNA processing protein A	LU					
	Cj0635 , holiday junction resovase	L					
	Cj0636, NOL-1/NOP-2/sun family protein	J					
	Cj0637, mrsA , putative peptide methionine sulfoxide reductase	O		truncated			truncated
cluster *Cj0643-0645*	*Cj0642, recN* , putative DNA repair protein	L					
	Cj0643, cbrR , two component response regulator	T					
	Cj0644, putative TatD-related deoxyribonuclease protein	L					
	Cj0645, putative secreted transglycosylase	M					
cluster *Cj0665-0694*	*Cj0665, argG* , argininosuccinate synthase	E					
	Cj0668, putative ATP/GTP-binding protein	R					
	Cj0669, ABC-transporter ATP-binding protein	R					
	Cj0670 , *rpoN* , RNA polymerase sigma-factor 54	K					
	Cj0671 , *dcuB* , anaerobic C4-dicarboxylate transporter	R					
	potassium-transporting ATPase A	-					
	Cj0677 , *kdpB* , potassium-transporting ATPase B	P					
	potassium-transporting ATPase C	-					
	Cj0679 , *kdpD* , truncated kdpD protein	T					
	Cj0680 , *uvrB* , excinuclease ABC subunit B	L					
	Cj0683 , putative periplasmic protein	-					
	Cj0684 , *priA* , putative primosomal protein N	L					
	Cj0685 , *cipA* , invasion protein	-					
	Cj0686, ispG , 4-hydroxy-3-methylbut-2-en-1-yl diphosphate synthase	I					
	Cj0687, flgH , putative flagellar L-ring protein precursor	N					
	Cj0688, pta , putative phosphate acetyltransferase	C					
	Cj0689, ackA , acetate kinase	C					
	Cj0690, putative restriction/modification enzyme	V					
	Cj0691, putative membrane protein	-					
	Cj0692, putative membrane protein	-					
	Cj0693, mraW , S-adenosyl-methyltransferase	M					
	Cj0694, putative periplasmic protein	O					
cluster *Cj0773-0775*	*Cj0773* , putative binding protein dependent transport system permease protein	P					
	Cj0774 , ABC transport system ATP-binding protein	P					
	Cj0775 , *valS* , valyl-tRNA-synthetase	J					

	Cluster	COG	xy259 (h)	RB922 (ch)	6399 (f)	04197 (b)	04199 (b)
cluster *Cj0849-0855*	*Cj0849* , conserved hypothetical protein	-					
	Cj0850 , putative MFS (Major Facilitator Superfamily) transport protein	-					
	Cj0851 , putative intergral membrane protein	-					
	Cj0852, putative integral membrane protein	S					
	Cj0853 , *hemL* , glutamate-1-semialdehyde 2, 1-aminomutase	H					
	Cj0854 , putative periplasmic protein	-					
	Cj0855 , *folD* , bifunctional protein	H					
cluster *Cj0967-0975*	*Cj0967* , putative periplasmic protein	-					
	Cj0970 , hypothetical protein	-					
	Cj0971 , hypothetical protein	-					
	Cj0972 , hypothetical protein	-					
	Cj0975 , putative outer-membrane protein	U					
cluster *Cj0984-0985*	*Cj0984* , conserved hypothetical protein	S					
	Cj0985 , *hipO* , hippurate hydrolase	R					
cluster *Cj1045-1051*	*Cj1045, thiG* , thiazole biosynthesis protein	H					
	Cj1046, moeB , putative molybdopterin biosynthesis protein	H					
	Cj1048 , *dapE* , succinyl-diaminopimelate desuccinylase	H					
	Cj1049, putative LysE family transporter protein	E					
	Cj1050, npdA , NAD-dependent deacetylase	K					
	Cj1051, cjeI , restriction modification enzyme	V					
cluster *Cj1061-1082*	*Cj1061, ileS* , isoleucyl-tRNA-synthetase	J					
	Cj1062, putative CinA-like protein	R					
	Cj1063 , putative acetyltransferase	E					
	Cj1066 , nitroreducatase family protein	C					
	Cj1067 , *pgsA* , CDP-diacylglycerol-glycerol-3-phosphate 3-phosphatidyltransferase	I					
	Cj1068 , putative peptidase M50 family	M					
	Cj1070 , *rpsF* , ribosomal protein S6	J					
	Cj1071 , *ssb* , single-strand DNA binding protein	L					
	Cj1073 , *lon* , ATP dependent protease La	O					
	Cj1074, putative lipoprotein	R					
	Cj1075 , hypothetical protein	S					
	Cj1076 , pyrroline-5-carboxylate reductase proC	E					
	Cj1077 , *ctsT* , putative periplasmic protein	-					
	Cj1079 , putative periplasmic protein	-					
	Cj1080, hemD , uroporphyrinogen-III synthase	-					
	Cj1081, thiE , tiamine-phosphate pyrophosphorylase	H					
	Cj1082, thiD , phosphomethylpyrimidine kinase	H					
cluster Cj1157-1171	*Cj1157, dnaX* , DNA polymerase III subunit gamma	L					
	Cj1161 , putative cation-transporting ATPase	P					
	Cj1163, putative cation transport protein	P					
	Cj1165, putative intergral membrane protein	S					
	Cj1166, putative intergral membrane protein	S					
	Cj1167 , *ldh* , L-lactate dehydrogenase	C					
	Cj1168, putative integral membrane protein (dedA homolog)	S					
	Cj1170, 50kda outer membrane protein precursor, omp50	-					
	Cj1171, ppi , peptidyl-prolyl cis-trans isomerase	O					
	Cj1172, conserved hypothetical protein	S					
cluster *Cj1178-1204*	*Cj1178,* highly acidic protein	-					
	Cj1179, fliR , flagellar biosynthetic protein	NU					
	Cj1180, ABC transporter, ATP-binding protein	V					
	Cj1181, tsf , elongation factor TS	J					
	Cj1182 , *rpsB* , 30S ribosomal protein S2						
	Cj1183, cfa , cyclopropane fatty-acyl-phospholipid synthase	M					
	Cj1184, petC , putative ubiquinol-cytochrome C reductase	C					
	Cj1185, petB , putative ubiquinol-cytochrome C reductase	C					
	Cj1186, petA , putative ubiquinol-cytochrome C reductase	C					
	Cj1187 , *arsB* , arsenical pump membrane protein	P					
	Cj1188 , *digA* , tRNA uridine 5-carboxymethylaminomethyl modification enzyme	D					
	Cj1189 , *cetB* , bipartite energy taxis response protein	T					
	Cj1190, cetA , bipartite energy taxis response protein	NT					
	Cj1191 , putative PAS domain containing signal-transduction sensor protein	T					
	Cj1192 , *dctA* , putative C4-dicarboxylate transport protein	C					
	Cj1193, putative periplasmic protein	-					
	Cj1194 , possible phosphate permease	P					
	Cj1195, pyrC2 , putative dihydroorotase	F					
	Cj1196, gpsA , glycerol-3-phosphate dehydrogenase [NAD(P)+]	C					
	Cj1197, gatB , aspartyl/glutamyl-tRNA(Asn/Gln) amidotransferase	J					
	Cj1198, luxS , S-ribosylhomocysteine lyase (autoinducer-2 production protein LuxS)	T					
	Cj1199 , putative iron/ascorbate-dependent oxidoreductase	R					
	Cj1200 , lipoprotein, NLPA family	P					
	Cj1201 , *metE* , 5-methyltetrahydropteroyltriglutamate-homocysteine methyltransferase	E					
	Cj1202, metF , 5,10-methylenetetrahydrofolate reductase	E					
	Cj1204 , *atpB* , ATP synthase F0, A subunit	C					
cluster *Cj1252-1259*	*Cj1252,* putative periplasmic protein	M					
	Cj1253, pnp , polyribonucleotide nucleotidyltransferase	J					
	Cj1256 , putative membrane protein	-					
	Cj1257, putative efflux pump	G					
	Cj1258 , putative phosphotyrosine protein phosphatase	T					
	Cj1259, porA , major outer membrane protein	-					
	Cj1260, dnaJ , chaperone	O					

	Cluster	COG	xy259 (h)	RB922 (ch)	6399 (f)	04197 (b)	04199 (b)
cluster *Cj1313-1334*	*Cj1313, pseH* , N-acetyltransferase,UDP-4-amino-4,6-dideoxy-beta-L-AltNAc	J					
	Cj1314, hisF , imidazole glycerol phosphate synthase subunit HisF	E					
	Cj1315, hisH , imidazole glycerol phosphate synthase subunit HisH	E					
	Cj1316, pseA , pseudaminic acid biosynthesis PseA protein	D					
	Cj1317, pseI , neuB, Pse synthetase	M					
	Cj1318, maf1 , motility accessory factor	S					
	Cj1319 , putative nucleotide sugar dehydratase	MG					
	Cj1320 , putative aminotransferase (degT family)	M					
	Cj1321 , putative transferase	R					
	Cj1322 , hypothetical protein	I					
	Cj1323 , hypothetical protein	-					
	Cj1324 , hypothetical protein	D					
	Cj1327 , *neuB2* , N-acetylneuraminic acid synthetase	M					
	Cj1328 , *neuC2* , putative UDP-N-acetylglucosamine 2-epimerase	M					
	Cj1329 , putative sugar-phosphate nucleotide transferase	MJ					
	Cj1330 , hypothetical protein	R					
	Cj1331 , *ptmB* , acylneuraminate cytidylyltransferase	M					
	Cj1332, ptmA , putative oxidoreductase	IQR					
	Cj1333, pseD , PseD protein	S					
	Cj1334, maf3 , motility accessory factor	S					
cluster *Cj1377-1388*	*Cj1377,* putative ferredoxin	C					
	Cj1378, selA , L-seryl-tRNA(SeC) selenium transferase	E					
	Cj1379, selB , putative selenocysteine-specific elongation factor	J					
	Cj1380 , putative periplasmic protein	O					
	Cj1381, putative lipoprotein	-					
	Cj1382, fldA , flavodoxin	C					
	Cj1383, hypothetical protein	-					
	Cj1384 , hypothetical protein	S					
	Cj1385 , *katA* , catalase	P					
	Cj1386 , ankyrin-repeat containing protein	R					
	Cj1387 , helix-turn-helix containing protein	S					
	Cj1388 , putative endoribonuclease L-PSP	J					
cluster *Cj1398-1400*	*Cj1398, feoB* , ferrous iron transport protein	P					
	Cj1399, hydA2 , putative Ni/Fe hydrogenase small subunit	C					
	Cj1400, faBI , putative enoyl-acyl-carrier-protein reductase NADH	I					
cluster *Cj1404-1412*	*Cj1404, nadD* , putative nicotinate-nucleotide adenylyltransferase	H					
	Cj1405 , conserved hypothetical protein	S					
	Cj1406 , putative periplasmic protein	-					
	Cj1407 , putative phospho-sugar mutase	G					
	Cj1408, fliL , putative flagellar protein	N					
	Cj1409, acpS , holo (acyl-carrier-protein) synthase	I					
	Cj1410 , putative membrane protein	-					
	Cj1411 , putative cytochrome P450	Q					
	hypothetical protein	-					
cluster *Cj1414-1418*	*Cj1414,* capsule polysaccharide modification protein	M					
	Cj1415, sylC , adenylylsulfate kinase,	P					
	Cj1416 , suger nucleotidyltransferase	M					
	Cj1417 , putative amidotransferase	R					
	Cj1418 , putative transferase	G					
cluster *Cj1455-1457*	*Cj1455, prfB* , peptide chain release factor 2	J					
	Cj1457, truD , tRNA pseudouridine synthase D	S					
cluster *Cj1481-1482*	*Cj1481* , putative helicase	L					
	Cj1482 , hypothetical protein	L					

	strains	*Cj0617*	*Cj1305*	*Cj1310*	*Cj1342*	*Cj1295*	*Cj1296*	*Cj1325*	*Cj1144*
**human**	xy259	G (10) - ON	**absent**	n.t.	G (9) - ON	G (9) - ON	G (10) - ON	G (10) - ON	11 (A), 9 (G) - OFF
	A588	G (9) - ON	G (10) - ON	G (9) - ON	G (9) - ON	G (10) - OFF	G (12) - OFF	G (10) - ON	12 (A), 9 (G) - OFF
	04410	G (9) - ON	G (8) - OFF	G (9) - ON	G (9) - ON	n.t.	n.t.	G (10) - ON	13 (A), 9 (G) - ON
	04293	n.t.	n.t.	n.t.	n.t.	n.t.	n.t.	G (10) - ON	11 (A),10 (G) - OFF
**chicken**	RB922	G (10) - ON	G (9) - ON	G (9) - ON	G (9) - ON	G (9) - ON	G (9) - OFF	G (10) - ON	11 (A), 9 (G) - OFF
	RB923	G (10) - ON	n.t.	G (9) - ON	G (9) - ON	G (9) - ON	G (10) - ON	G (10) - ON	11 (A),10 (G) - OFF
**duck**	6812	G (9) - ON	G (10) - ON	G (8) - OFF	G (9) - ON	n.t.	n.t.	n.t.	13 (A), 9 (G) - ON
**bovine**	04197	n.t.	n.t.	n.t.	n.t.	n.t.	n.t.	G (10) - ON	13 (A), 8 (G) - OFF
	04199	n.t.	n.t.	n.t.	n.t.	n.t.	n.t.	G (9) - OFF	10 (A), 9 (G) - ON
**milk**	6399	G (11) - OFF	G (8) - OFF	G (9) - ON	G (10) - OFF	G (9) - ON	G (10) - ON	G (9) - OFF	9 (A), 9 (G) - OFF
	7731	G (9) - ON	G (9) - ON	G (9) - ON	G (9) - ON	n.t.	n.t.	G (10) - ON	14 (A),10 (G) - OFF
	7732	G (9) - ON	G (10) - ON	G (9) - ON	G (9) - ON	n.t.	n.t.	G (10) - ON	14 (A),10 (G) - OFF
	6278	n.t.	n.t.	n.t.	n.t.	n.t.	n.t.	n.t.	13 (A), 8 (G) - OFF

Table S7.: Variability in nucleotide repeats in phase-variable genes of the flagellar glycosylation locus. The repeat lengths were re-analyzed and confirmed for all genome sequenced strains and other selected ST-21 strains by Sanger sequencing. Cj numbers represent gene numbering in reference strain 11168 (Parkhill et al., 2000)

Intergenic regions	strains	comments
Cj1253 (pnp) - Cj1256	04199	deletion of ca 200 bp
Cj1258 - Cj1259 (porA)	04199	sequence variability
Cj1259 - Cj1260 (dnaJ)	04199 + 04197	sequence variability
Cj1193 - Cj1194	04199 + 6399	sequence variability
Cj1192 (dctA) - Cj1191	04199 + 04197	sequence variability
Cj1183 (cfa) - Cj1182 (rpsB)	04199 + 04197	sequence variability
Cj0967 - Cj0970	04199	repeat variability
Cj0987 - Cj0990	04199 + 6399	repeat variability
Cj0017 (dsbl) - Cj0019	04199 + 6399	sequence variability
Cj0020 - Cj0021	04197	insertion of ca 60 bp
Cj0025 - Cj0026 (thyX)	04199 + 04197	sequence variability
Cj0167 - Cj0169 (sodB)	04197	sequence variability
Cj1519 (moeA2) - Cj1521	04199+04197+6399	insertion of different numbers of bp
Cj0566 - Cj0569	04199	repeat variability

Table S8: List of nucleotide variation and polymorphisms in intergenic regions in the five ST-21 genome sequences analyzed using one alignment (in Kodon) of the five genomes with the 04199 genome as reference. Gene nomenclature is derived from gene numbers of one *C. jejuni* reference strain 11168 (Parkhill et al., 2000) used for the gene annotation. Variation of intergenic regions was confirmed by PCR and Sanger sequencing.

strains	sources	group	Fbox	TIGR	Tox	Redox
A 588	human	gr 1 ST-21	red	green	green	red
xy 259			green	green	red	green
04410			red	red	red	red
04293			red	red	red	red
04299			red	red	green	red
04438			red	red	red	red
04448			red	red	green	red
6399	milk		red	red	red	red
7731			red	red	red	red
7732			red	red	red	red
7928			red	red	green	red
6660			red	red	green	red
6661			red	red	green	red
6278			red	red	red	red
7239			red	red	red	red
7255	poultry		red	red	green	red
7345			red	red	red	red
RB 922	chicken		red	green	green	red
RB 923			red	green	green	red
04197	bovine		red	green	green	red
04199			red	red	red	red
6812	duck		red	red	red	red
6917	lamb		red	red	green	red

Table S9.: Distribution of selected phage-related genes (gene names see also Supplementary Table S5, marked by asterisks) within ST-21 strains from different sources. Genes were detected by PCR with gene-specific primers. Color-coding of genes: green: gene present; red: gene not present.

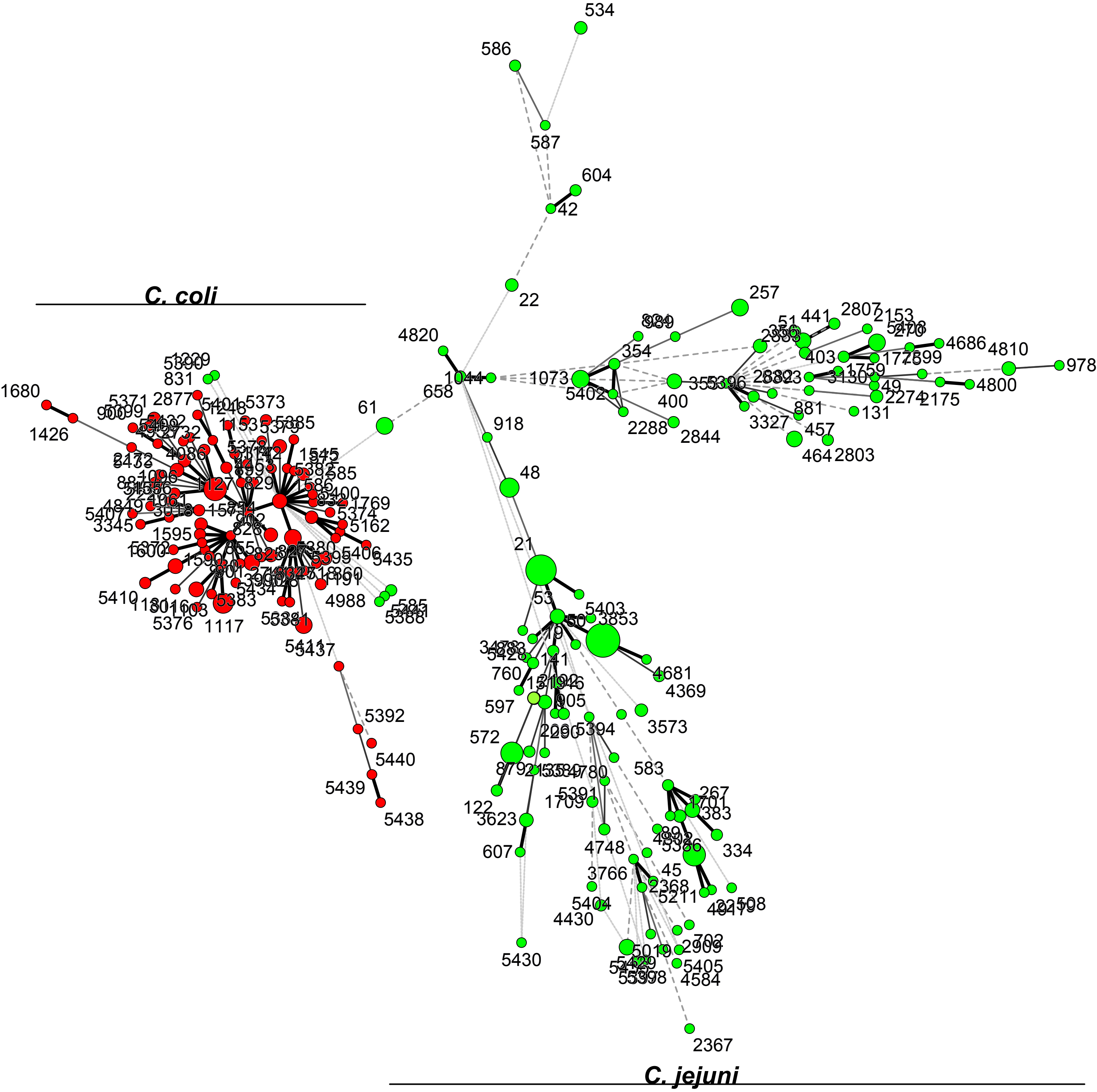

Gripp et al., Fig. S1:
Minimal spanning tree of all FBI-Zoo *Campylobacter* isolates colour-coded according to their assignment to the two *Campylobacter* species (*C. coli*-red and *C. jejuni*-green). Several strains which are assigned to the other species are visible in the *C. coli* clade (red bush). All STs are indicated by numbers.

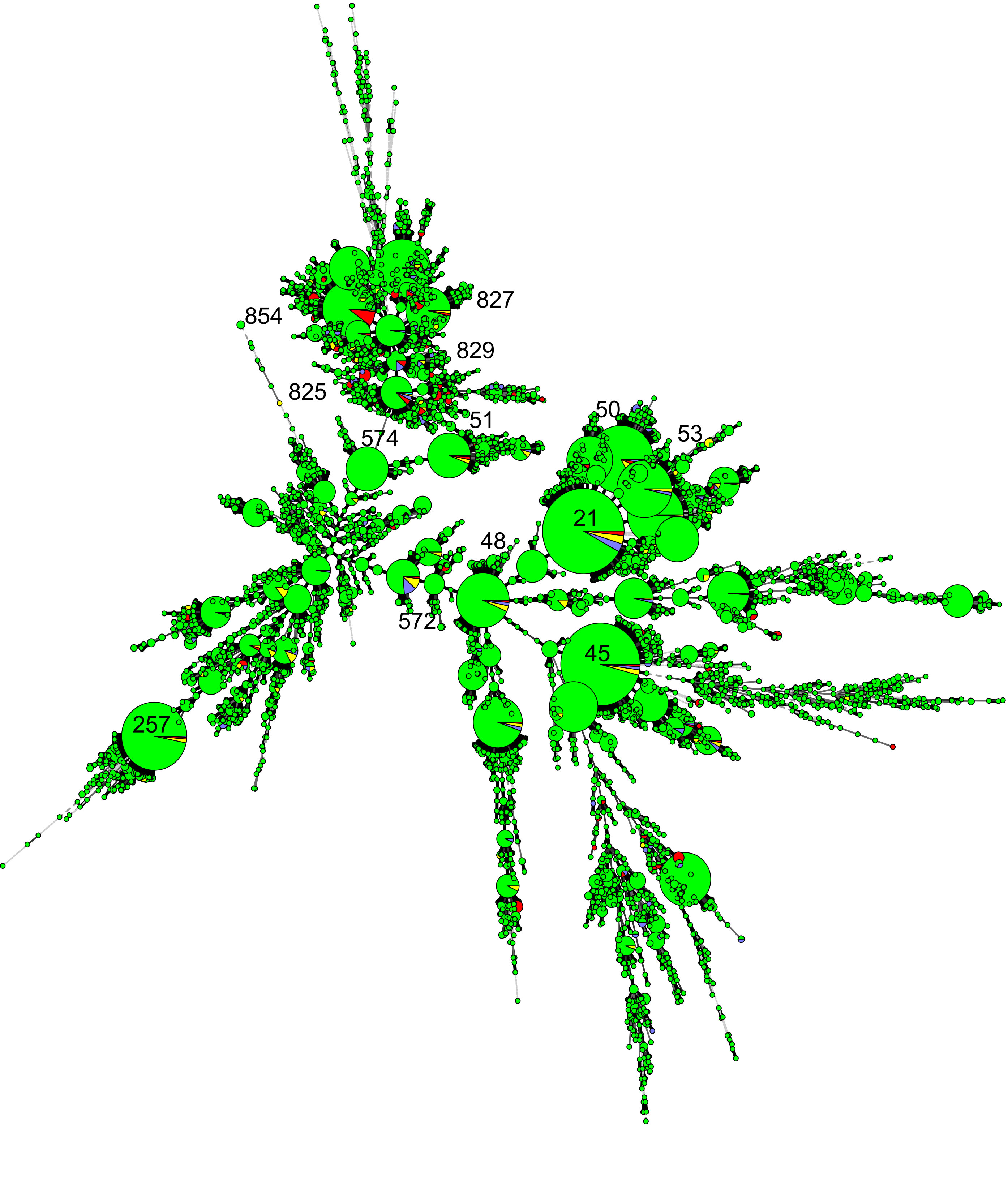

Gripp et al., Fig. S2
Minimal spanning tree of all FBI-Zoo *Campylobacter* isolates (colouring: blue-human, yellow-animal and red-food) in the context of all strains of the PubMLST database (PubMLST strains shown in green; numbers as of June 2011). Dominant STs are indicated by numbering.

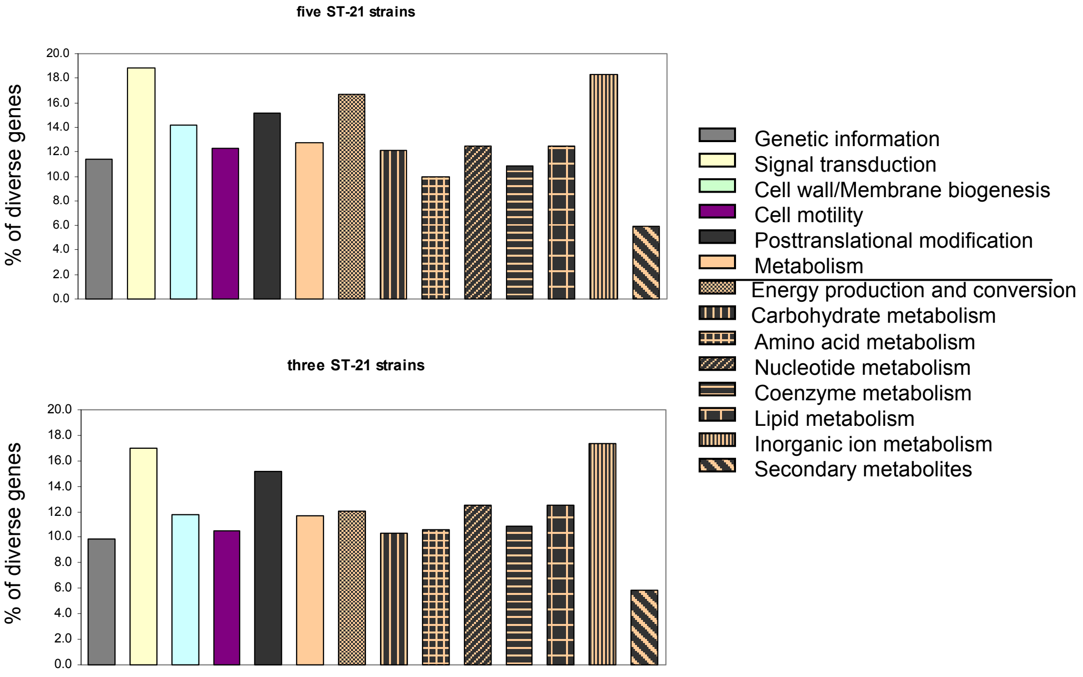

Gripp et al., Fig. S3.: **Gene categories (COG) in recombined stretches of sequence in ST-21 *C. jejuni* genome sequences.** The assignment of genes affected by recombination into functional groups was based on the COG classification for strain NCTC 11168. The genome sequences of all five strains (xy259 (h), RB922 (ch), 6399 (f), 04197 (b) and 04199 (b)) analyzed in the upper panel and of the three strains (of milk and bovine origin) analyzed in the lower panel were grouped according to COG gene functions of all genes within clusters of genes showing recombination events. Recombination-affected genes belonging to more than one functional group were counted once for each category. The COG functional group "metabolism" was further subdivided into eight defined COG subcategories as indicated by different hatched bars. The ratio of numbers of affected genes in the COG categories and all genes in the same categories were then calculated as percent (percent of recombined genes in each category). The frequency differences in recombination events between the categories were not statistically significant. The basis for the graphics was the COG classification shown in Table S6.

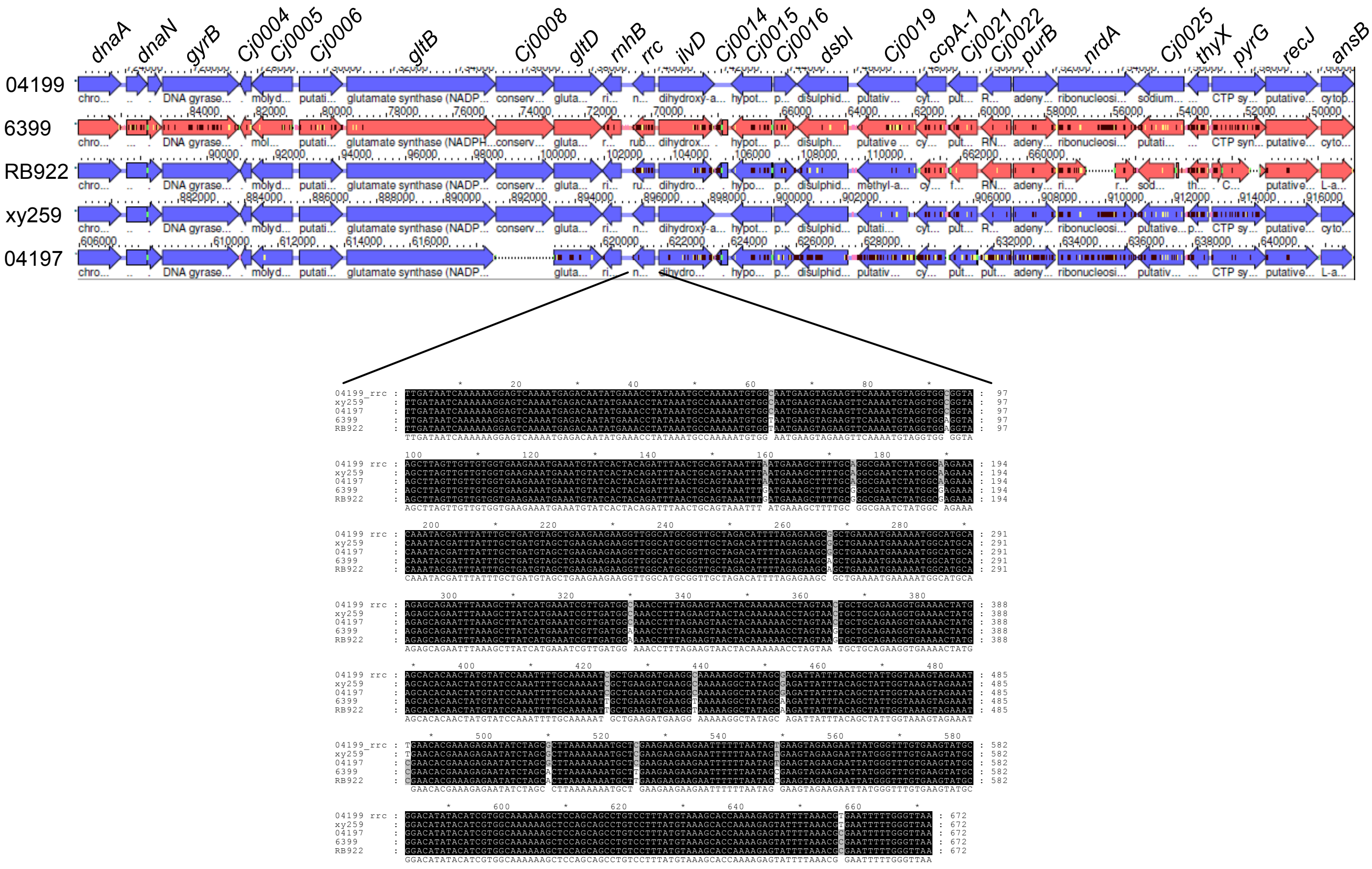

Gripp et al., Fig. S4.: **Kodon snapshot from the alignment of five ST-21 strains with 04199 as reference.** The recombined cluster in the region from *dnaA* (*Cj0001*) to *ansB* (*Cj0029*), also shown in Table S6, was chosen to visualize the aspects of recombined sequences. Color-coding of polymorphisms indicated by vertical lines in the upper panel: pink – intergenic mutations, black – silent mutations, yellow – missense mutations. In the lower panel, the DNA sequence of gene *rrc* (*Cj0012,* non-haem iron protein) is shown as a representative example to illustrate the extent of DNA polymorphisms between strains. Notes: *dnaN* in the strain 04199 has a repeat length variability leading to a frameshift (not re-resequenced); *Cj0008* is absent in 04197; *Cj0014* is not annotated in 04199 and xy259 due to deletion of 128 bp; the genes *nrdA* and *pyrG* in RB922 are divided due to contig ends.
